# A Genome-Wide Association Study of the Maize Hypersensitive Defense Response Identifies Genes That Cluster in Related Pathways

**DOI:** 10.1371/journal.pgen.1004562

**Published:** 2014-08-28

**Authors:** Bode A. Olukolu, Guan-Feng Wang, Vijay Vontimitta, Bala P. Venkata, Sandeep Marla, Jiabing Ji, Emma Gachomo, Kevin Chu, Adisu Negeri, Jacqueline Benson, Rebecca Nelson, Peter Bradbury, Dahlia Nielsen, James B. Holland, Peter J. Balint-Kurti, Gurmukh Johal

**Affiliations:** 1 Department of Plant Pathology, NC State University, Raleigh, North Carolina, United States of America; 2 Department of Botany and Plant Pathology, Purdue University, Lilly Hall, West Lafayette, Indiana, United States of America; 3 Department of Plant Pathology and Plant-Microbe Biology, Cornell University, Ithaca, New York, United States of America; 4 Institute for Genomic Diversity, Cornell University, Ithaca, New York, United States of America; 5 Department of Biological Sciences, NC State University, Raleigh, North Carolina, United States of America; 6 USDA-ARS Plant Science Research Unit, Raleigh, North Carolina, United States of America; 7 Department of Crop Science, NC State University, Raleigh, North Carolina, United States of America; Virginia Tech, United States of America

## Abstract

Much remains unknown of molecular events controlling the plant hypersensitive defense response (HR), a rapid localized cell death that limits pathogen spread and is mediated by resistance (R-) genes. Genetic control of the HR is hard to quantify due to its microscopic and rapid nature. Natural modifiers of the ectopic HR phenotype induced by an aberrant auto-active R-gene (*Rp1-D21*), were mapped in a population of 3,381 recombinant inbred lines from the maize nested association mapping population. Joint linkage analysis was conducted to identify 32 additive but no epistatic quantitative trait loci (QTL) using a linkage map based on more than 7000 single nucleotide polymorphisms (SNPs). Genome-wide association (GWA) analysis of 26.5 million SNPs was conducted after adjusting for background QTL. GWA identified associated SNPs that colocalized with 44 candidate genes. Thirty-six of these genes colocalized within 23 of the 32 QTL identified by joint linkage analysis. The candidate genes included genes predicted to be in involved programmed cell death, defense response, ubiquitination, redox homeostasis, autophagy, calcium signalling, lignin biosynthesis and cell wall modification. Twelve of the candidate genes showed significant differential expression between isogenic lines differing for the presence of *Rp1-D21*. Low but significant correlations between HR-related traits and several previously-measured disease resistance traits suggested that the genetic control of these traits was substantially, though not entirely, independent. This study provides the first system-wide analysis of natural variation that modulates the HR response in plants.

## Author Summary

The hypersensitive pathogen defense response (HR) in plants typically consists of a rapid, localized cell death around the point of attempted pathogen penetration. It is found in all plant species and is associated with high levels of resistance to a wide range of pathogens and pests including bacteria, fungi, viruses, nematodes, parasitic plants and insects. Little is known about the control of HR after initiation, largely because it is so rapid and localized and therefore difficult to quantify. Here we use a mutant maize gene conferring an exaggerated HR to quantify HR levels in a set of 3,381 mapping lines characterised at 26.5 million loci to identify genes associated with naturally-occurring variation in HR. Many of these genes seem to be involved in a set of connected regulatory pathways including protein degradation, control of programmed cell death, recycling of cellular components and regulation of oxidative stress. We have also shown that several of these genes show high levels of expression induction during HR. The study provides the first comprehensive list of natural variants in maize genes that modulate HR and cluster within reported pathways underlying molecular events during HR.

## Introduction

Programmed cell death (PCD) can be defined as death of a cell mediated by intracellular signaling [1]. Apoptosis, the most common form of PCD in animals, is defined by certain hallmark characteristics including cellular shrinkage, ‘blebbing’ and nuclear fragmentation [2]. The other major form of programmed cell death identified in animals is autophagic cell death which has been defined as “a type of cell death occurring together with (but not necessarily by) autophagic vacuolization” [3]. Autophagy itself is the degradation of components of the cell through the action of lysozomes. As well as being involved in PCD, in many cases autophagy is regarded as a ‘pro-survival’ response, allowing cells to survive various types of stress [4].

PCD is perhaps best thought of as a complex of related processes and is important in many developmental processes in plants including leaf senescence, degeneration of cereal aleurone cells, development of tracheary elements in xylogenesis, and cell death in plant reproduction [5]. There is some discussion in the literature regarding the relationship between animal and plant PCD mechanisms. A recent review suggested that animal and plant PCD are not analogous and that two major types of plant PCD should be recognized: vacuolar cell death, characterized by the removal of cell contents by autophagy and the release of hydrolases from lytic vacuoles, and necrosis, characterized by early rupture of the plasma membrane and shrinkage of the protoplast [6].

The plant hypersensitive response (HR) is a key immune response of plants that confers resistance to almost every type of pathogen; bacteria, viruses, fungi, nematodes, insects and even parasitic plants [7]. HR is a form of PCD characterized by rapid, localized cell death at the point of attempted pathogen penetration, usually resulting in disease resistance [8]. It is often associated with other responses, including ion fluxes, an oxidative burst, lipid peroxidation and cell wall fortification [9]. Van Doorn et al [6] suggest that HR is a type of PCD sharing features with, but distinct from, both vacuolar cell death and necrosis.

HR is generally effective against biotrophic pathogens, since biotrophs require a long-term feeding relationship with living cells of the host. It is generally mediated by dominant plant resistance genes (R-genes) whose activation is triggered by the direct or indirect detection of specific pathogen effector proteins [10]. Crucially, R-proteins are maintained in their inactive state if their corresponding effector is not present. Mutants in which HR is constitutively active have been identified in many plant species, including maize [11], [12], Arabidopsis thaliana [13], barley (*Hordeum vulgare*) [14], and rice (*Oryza sativa*) [15].

Plant lesion mimics mutants, or lesioned mutants are a class of mutants that spontaneously form lesions (patches of dead cells) in the absence of any obvious injury, stress or infection to the plant. Since these lesions in some cases resemble HR or lesions casued by disease these mutaions have been termed disease lesion mimics [16]. In fact many of the genes underlying this mutant class are likely not involved in defense response pathways but are components of various pathways, all of which cause cell death if their function is perturbed [11]. For instance the *Arabidopsis* gene *acd*2 and the maize gene *lls*1 are defective in chlorophyll degradation [17], [18]. However several lesion mimic genes are indeed involved in the defense response in their wild type form. One such gene is derived from the *Rp1* locus of maize.

The *Rp1* locus on maize chromosome 10 carries multiple tandemly-repeated *R*-gene paralogs with characteristic coiled-coil, nucleotide-binding site and leucine-rich repeat (CC-NBS-LRR) domains, some of which confer resistance to specific races of maize common rust conferred by the fungus *Puccini sorghi* [19]. The locus is meiotically unstable due to a high frequency of unequal crossovers between paralogs [20]. Unequal crossing over followed by intragenic recombination resulted in the formation of the chimeric gene *Rp1-D21* [21], [22]. In the *Rp1-D21* protein, the recognition and elicitation functions are uncoupled, causing the spontaneous activation and formation of HR lesions on the leaves and stalks of the plant in the absence of pathogen recognition. *Rp1-D21* has partially dominant gene action and its phenotypic effect is influenced by the environment, developmental stage and genetic background [21], [23]–[26]. In this and in past studies [23]–[25], [27] we make the explicit assumption that the cell death caused by *Rp1-D21* is an exaggerated form of the HR that is, in normal circumstances, a response to pathogenesis. This assumption is based on a number of lines of reasoning: Most importantly Rp1-D21 is a typical CC-NB-LRR protein of the type that is often associated with HR-mediated resistance [10]. *Rp1-D* and several other alleles confer pathogen-dependent HR in maize and transgenic expression of Rp1-D21 confers a constitutive HR phenotype in maize [21]. Furthermore, we have shown that the cell death conferred by *Rp1-D21* is associated with typical hallmarks of HR including the accumulation of salicylic acid (G-F Wang unpublished) and reactive oxygen species and the expression of pathogenesis-related genes [23], [25]. Finally *Rp1-D21* mediated HR is temperature and light-dependent dependent [25], which is typical of HR associated with R-genes [25], [28]–[30].

Since the *Rp1-D21* HR phenotype is an exaggerated defense response [23], it is likely that many or all of the genes that modify the aberrant *Rp1-D21*-associated HR are also associated with variation in the wild type defense response. Thus the *Rp1-D21* phenotype can be used as a reporter for the identification of loci affecting the strength and severity of HR. This approach, in which a mutant phenotype is used as a reporter to reveal normally undetectable genetically-controlled variation, has been termed Mutant-Assisted Gene Identification and Characterization (MAGIC) [31].

In previous work [23], [24], [27], a maize inbred line (H95) into which *Rp1-D21* was introgressed and maintained in a heterozygous condition (designated *Rp1-D21-*H95) was crossed with lines from several mapping populations. By phenotyping the resulting F_1_ families, several quantitative trait loci (QTL) modulating the HR conferred by *Rp1-D21* were identified. A genome-wide scan of HR phenotypes scored on progenies of crosses between the heterozygous *Rp1-D21* tester line and 231 diverse inbred lines of maize (constituting a high-resolution association mapping panel) identified six significantly associated SNPs, five of which are located within or immediately adjacent to candidate causative genes predicted to play significant roles in the control of programmed cell death and especially in ubiquitin pathway-related genes [27].

Here we have significantly expanded and refined this analysis by crossing *Rp1-D21-*H95 into a large sample of the maize nested association mapping (NAM) population, a 5000-member recombinant inbred line (RIL) population derived from 25 diverse parents [32]. Taking advantage of the power of joint linkage analysis and high-resolution of genome-wide association mapping in this population, we were able to characterize the genetic architecture controlling the maize HR in a comprehensive fashion. We identified a set of candidate genes implicating several regulatory pathways in controlling HR. These include mechanisms that regulate protein degradation, oxidative stress, lignin biosynthesis, PCD and autophagy. We have further shown that the expression of many of these genes is upregulated in the presence of the *Rp1-D21* gene.

## Results

### Evaluation of HR-related phenotypes and correlation with disease resistance traits

The heritability estimates for the measured traits, lesion severity (LES), height ratio (HTR) and stalk width ratio (SWR) were all high, with line mean basis heritability ranging from 0.83 to 0.87, while for days to anthesis ratio (DTAR) it was 0.58 (Table S1). Line within population, population-by-environment interaction, and line-by-environment interaction within population were all significant contributors to variance for all traits. Figure S1 shows the distribution of phenotypic values across the subpopulations. The *Rp1-D21*-H95 mutant line which was crossed to the NAM recombinant inbred lines (RILS) to generate the F1 families that were scored, had a relatively severe HR phenotype with scores of 7.3, 0.47, 0.39 and 0.95 for LES, HTR, SWR and DTAR, respectively (for comparisons see Fig. S1).

### NAM linkage, QTL and GWA analysis

**Figure 1:**
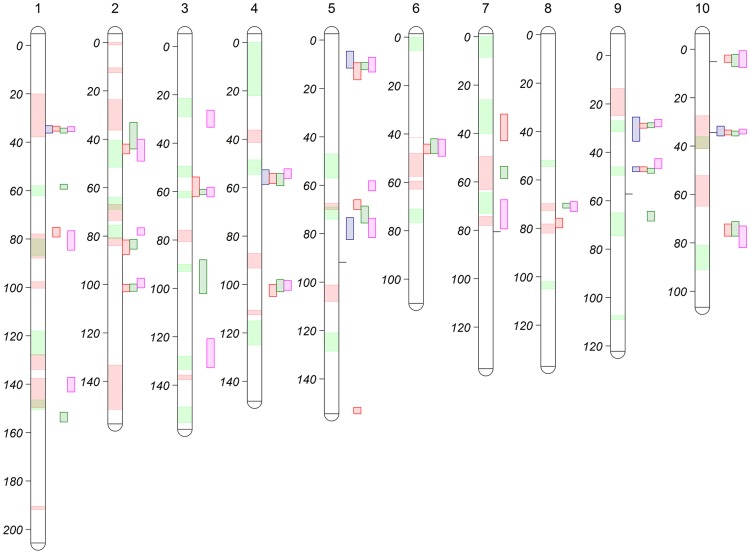
NAM joint-linkage QTL analysis for HR-related traits (LES, HTR, SWR and DTAR) across all the 10 maize chromosomes/linkage groups. Units on vertical bars are in centi-Morgan (cM) genetic distance. Colored bars on right side of chromosome bar indicate the HR-related trait QTL support intervals: purple- DTAR; red-HTR; green-LES pink-SWR, while green and red segments inside chromosome bars correspond to SLB and NLB resistance QTL support intervals, respectively (Derived from KUMP et al. [34]; POLAND et al. [35]). Horizontal lines on the right side of chromosomes 5, 7,9,10 indicate significant SNP hits for HR-related traits from a previous study (OLUKOLU et al. [27]).

**Figure 2:**
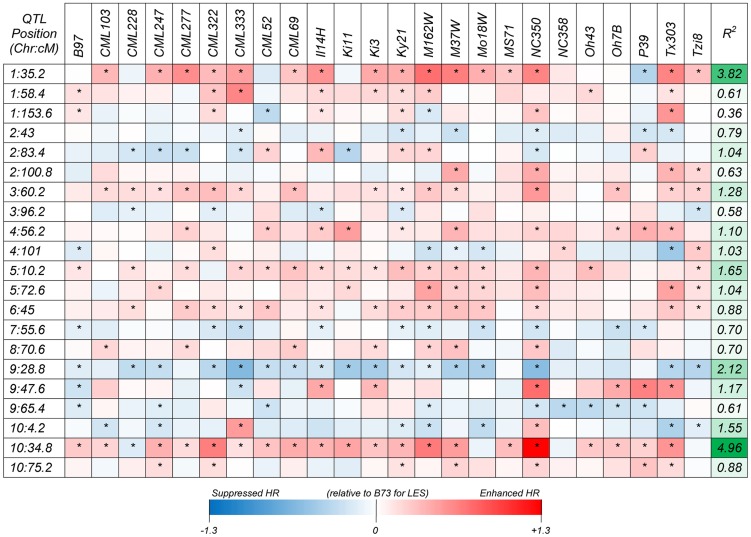
Heat map showing additive allelic effects for the HTR across 24 NAM founder lines relative to the common B73 parent. Chromosome and genetic map positions (centiMorgans; cM) of QTL peaks are shown on the left vertical axis, the contribution to variance among RIL mean values across all 24 NAM populations are shown on right vertical axis and the NAM founder lines are shown on the horizontal axis. Scale below heat map indicates range of allelic effect values and corresponding color intensity. Boxes with asterisks indicate significant (*p*<0.05) allelic effects.

**Figure 3:**
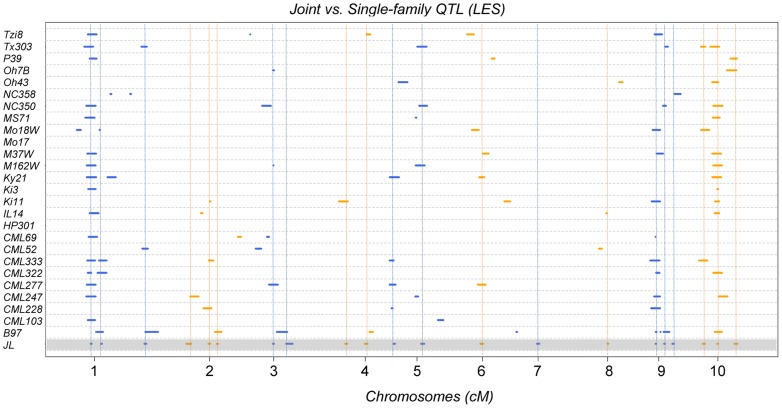
LES QTL obtained from single and joint-linkage QTL analysis across all the 10 maize chromosomes/linkage groups. Parental inbred lines crossed with the common B73 inbred line to derive each bi-parental sub-population are shown on the vertical axis. The results of joint linakage analysis across the NAM population comprising 24 populations is indicated as JL. The genetic distance for each chromosome is represented in cM (centi-Morgan) unit on the horizontal axis. Different colours are used to indicate alternating chromosomes.

Following a joint family stepwise regression analysis of the HR-related traits, 21, 19, 22 and 7 QTL were detected for LES, HTR, SWR and DTAR, respectively (Fig. 1 and File S1). Altogether, 32 distinct non-overlapping QTL regions were identified. A total of six QTL overlapped across all four traits while 14 were unique to one trait (Fig. 1). For all four traits, two QTL, on chromosome 1 (QTL peak at about 35 cM) and 10 (QTL peak at about 34.5 cM) explained a high proportion of the phenotypic variance and showed high allelic effects in most of the 24 NAM sub-populations (Fig. 2, Fig. S2 and File S2). QTL on chromosomes 3, 5, 6, 9 and 10 with peaks at about 60, 11, 46, 29 and 4 cM, respectively, had significant allelic effects in more than half of the 24 NAM populations (Fig. 2, Fig. S2). Single family QTL analyses identified QTL with similar locations and effects as joint family QTL analysis, but identified more small effect QTL that were often specific to individual families (Fig. 3, Fig. S3). The major QTL on chromosome 1 (QTL peak at about 35 cM) and 10 (QTL peak at about 34.5 cM) accounted for as much as 35.3 and 38.5% of the phenotypic variation, respectively, within specific individual families (File S3).

Exhaustive two-dimensional searches for epistatic QTL interactions in the joint linkage model were performed for all four lesion mimic-derived traits. Only QTL interactions for LES, HTR and SWR passed the initial filter for putatively significant interactions (File 10). However, no epistatic QTL interactions were significantly associated (all *p*-values greater than permutation test-based thresholds) with traits when fit simultaneously with the additive joint linkage QTL.

Genome Wide Association (GWA) analysis identified more than one hundred SNPs with a resample model inclusion probability (RMIP) ≥0.25 (Table 1, Figure S4). In some cases, clusters of associated SNPs were identified within ∼100–2,000 bp of each other. In these cases we assumed that SNPs were all detected due to their linkage with the same causative gene and the SNP with the highest statistical significance was chosen as being representative of the cluster. Ultimately, 44 significantly associated SNPs, representing 44 distinct loci were identified in this way. Thirty-six of these SNPs were within 23 of the 32 non-overlapping QTL identifed by joint linkage analysis. Consistent with the QTL analysis, the most significant large effect QTL on chromsomes 1 and 10 harboured SNPs with lowest GWAS *p* values (as low as 1.89×10^−36^) and largest RMIP (as much as 0.95) and allelic effect values, indicating strong associations and higher contributions to the phenotype variance (Files S4,S5).

**Table 1:** SNPs significantly associated with HR-related traits (LES, HTR, SWR and DTAR), and closest predicted maize genes (more details can be found in File S5).

Chr^1^	Position (bp)^2^	*p-value* 3 *(−log10)*	RMIP^4^	Allele effect^5^/QTL^6^	Gene ID	Maize gene name or best matching ortholog^7^	DEG^8^		Functional annotation
							B73	Mo17	
***Candidate genes with significant differential expression between mutant Rp1-D21 and wild type (FDR <0.2)***									
1	17826971	33.3	0.95	+/4^9^	GRMZM2G061806	Hydroxycinnamoyl-CoA shikimate/quinate hydroxycinnamoyl transferase	296	224	LIG, DFR, CWM
10	21804495	21.5	0.66	+/4^9^	GRMZM2G109582	UEV domain/VPS23/ELC	8.4	6.9	AUT, mUBQ
5	4758329	13.9	0.22	+/4^9^	GRMZM2G028813	IQ calmodulin-binding motif domain containing protein	2.2	4.2	Ca^2+^Sig, DFR
9	16319620	8.4	0.67	−/4^9^	GRMZM2G099363	Caffeoyl-CoA O-methyltransferase	2.2	1.7	LIG, DFR, CWM
3	126183689	8.9	0.13	+/3^9^	GRMZM2G351387	Spotted leaf 11/plant U-box 13^7^	2.2	2.5	PCD, UBQ
10	3909109	11.0	0.53	−/3^9^	GRMZM2G061742	RP1/NB-ARC domain-containing disease resistance protein^7^	0.7	2.3	PCD, DFR
4	188208471	8.5	0.57	−/3	GRMZM2G439311	IQ calmodulin-binding motif domain containing protein	4.8	4.5	Ca^2+^Sig, DFR
2	194043588	13.3	0.54	+/3	GRMZM2G079231	Tetratricopeptide repeat (TPR)-like superfamily protein	1.8	2.4	CHP
7	144228691	8.8	0.27	−/1	GRMZM2G160922	Serine/threonine-protein kinase CTR1	4.2	5.4	PCD, DFR
7	121255571	11.8	0.22	−/1	GRMZM2G135763	Pectin lyase-like superfamily protein	1.9	2.2	DFR, CWM
1	16578218	8.5	0.49	−/0	GRMZM2G017616	Lipoxygenase 3	3.8	5.7	PCD, RxH
1	21469459	9.4	0.37	+/0	GRMZM2G004422	RING/U-box superfamily protein	2.3	2.9	PCD, UBQ
***Candidate genes with non-significant differential expression between mutant Rp1-D21 and wild type (FDR <0.2)***									
1	18100677	8.3	0.39	−/4^9^	GRMZM2G023575	Modifier of rudimentary (Mod(r)) protein, VPS37	1.3	1.3	AUT, mUBQ
5	3545354	11.6	0.25	+/4^9^	GRMZM2G318346	Cytochrome bd ubiquinol oxidase, 14kDa subunit	1.3	1.3	RxH
10	3276213	13.9	0.58	+/3^9^	GRMZM5G873586	Jacalin-like lectin domain containing protein/CRK7^7^	1.8	1.1	PCD, DFR
3	36813365	15.7	0.14	+/3^9^	GRMZM2G069009	Zinc finger C-x8-C-x5-C-x3-H type	1.2	1.1	DFR
10	3540773	11.5	0.55	−/3^9^	GRMZM2G069382	RP1/NB-ARC domain-containing disease resistance protein^7^	0.9	1.2	PCD, DFR
6	122660930	14.5	0.29	+/3^9^	GRMZM2G071100	Calmodulin-related calcium sensor protein, OsCML30/MSS3	0.8	0.9	Ca^2+^Sig
10	142092959	8.3	0.27	+/3	GRMZM2G119802	methyl-CPG-binding domain protein 13, MBD4	0.5	0.5	PCD
2	16634640	7.4	0.27	−/3	GRMZM2G080992	MATE efflux family protein, EDS5/SID1	0.7	1.6	RxH, DFR
8	138472371	10.4	0.52	+/2	GRMZM2G700683	Carbamoyl phosphate synthetase B	1.1	1.0	PCD
8	139341575	10.2	0.39	+/1	GRMZM2G027333	ZOS1-10 - C2H2 zinc finger protein/indeterminate(ID)-domain 7	0.5	0.5	DFR
5	216776356	9.8	0.24	+/1	GRMZM2G433025	Protein of unknown function (DUF177)	0.6	0.6	-
1	257206433	14.2	0.8	+/1	GRMZM2G050684	Cystathionine b-synthase (CBS) domain-containing protein	0.8	0.8	RxH
10	49011347	10.5	0.46	+/0	GRMZM2G350748	Guanylyl cyclase-like protein, SPK1	1.4	0.6	PCD
1	3644382	7.2	0.56	+/0	GRMZM2G044388	Cyclin-like F-box	0.7	0.7	PCD, UBQ
5	15725848	9.1	0.28	−/0	GRMZM2G100246	Transcription regulator NOT2/NOT3/NOT5 family protein	0.9	0.8	UBQ, RNAp
***Candidate genes without detectable unique transcript***									
5	2282955	7.2	0.39	+/4^9^	GRMZM2G144042	Protein Kinase 1b	-	-	DFR
9	16912715	8.2	0.33	−/4^9^	GRMZM2G002227	TPR repeat domain/STI-like/U-box domain-containing protein 33-like	-	-	CHP, STR
9	96001269	21.0	0.87	+/4	GRMZM2G145104	RING/U-box superfamily protein	-	-	PCD, UBQ
10	20799373	15.3	0.25	+/4^9^	GRMZM2G331368	ubiquitin-protein ligase 1/HECT domain	-	-	UBQ
10	21022896	28.3	0.75	+/4^9^	GRMZM2G141948	DNA polymerase I family protein	-	-	-
10	21788845	26.3	0.24	+/4^9^	GRMZM2G001500	CPHSC70-HEAT SHOCK PROTEIN	-	-	AUT, STR
10	25214435	35.7	0.53	+/4^9^	GRMZM2G303536	Thioredoxin superfamily protein	-	-	RxH
2	192263493	16.8	0.36	+/3	GRMZM2G129034	MADS-box transcription factor, OsMADS8	-	-	TF, LIG
2	170625752	12.4	0.68	−/2	GRMZM2G008313	Jacalin-like lectin domain containing protein^7^	-	-	PCD, DFR
5	81794295	9.6	0.26	+/2	GRMZM2G011888	Sugar isomerase (SIS) family protein	-	-	-
5	135538903	13.4	0.28	+/2	GRMZM2G128445	GNS1/SUR4 membrane protein family	-	-	-
5	146951564	16.3	0.39	+/2	GRMZM2G429958	PP2A-like family of phosphoprotein phosphatases/peptidase C48 domain	-	-	PCD, UBQ
5	147122610	15.0	0.48	+/2	GRMZM2G077313	Ulp1 protease family/ESD4-like sumo protease 1	-	-	UBQ
1	41106935	7.9	0.34	+/1	GRMZM2G428410	Expressed protein	-	-	-
1	295596386	7.7	0.27	−/0	AC177926.2_FGT003	Putative NAC domain transcription factor	-	-	TF
8	141135870	9.7	0.25	+/0	GRMZM2G021289	Plant thionin family protein precursor	-	-	DFR
8	166955466	7.3	0.34	−/0	GRMZM2G447876	Phosphatidyl serine synthase family protein	-	-	PCD

1 chromosome;

2 physical map position based on B73 maize reference genome v2;

3 −log10 of average *p* value from GWA analysis based on 100 resampling method;

4 resampling model inclusion probability (RMIP);

5 positive and negative allelic effects relative to B73 imply enhancing and suppressing effect on the HR phenotype, respectively;

6 Number of HR-related trait QTL colocalizing with associated SNP hits (maximum is 4);

7 mutations of these candidate genes induce lesion phenotypes;

8 differentially expressed genes (DEG), expression level ratios of candidate genes between mutant and wild type F1 plants from the cross B73×*Rp1-D21*-H95 and Mo17: *Rp1-D21*-H95.

9 Associated SNPs/candidate genes colocalized within QTL that were detected in more than 12 of the 24 NAM populations.

Key: Lignification: LIG; Programed cell death: PCD,; Autophagy, AUT; monoubiquitination, mUBQ; Calcium signaling, Ca^2+^Sig; defense response, DFR; redox homeostasis, RxH; ubiquitination, UBQ; CWM: cell wall modification; TF, transcription factor; STR: stress response; CHP: molecular chaperone; RNAp, RNA processing.

### Candidate genes, transcript expression levels and associated pathways

The closest predicted gene to each of the 44 SNPs was identified based on the publicly available maize genome (http://maizesequence.org, Table 1, File S5). Eleven of the 44 SNPs loci were within the coding region of the candidate genes, while the other 33 candidate genes were the closest predicted genes to each SNP, with physical map distances between the SNP and the candidate gene ranging from 33 bp to 83,325 bp (File S5).

Based on functional annotations of the candidate genes, we identified several groups of genes predicted to function in common pathways that have been associated with HR: PCD, autophagy, redox homeostasis, ubiquitin-mediated protein degradation, calcium signaling, lignin biosynthesis and the defense response (Table 1, File S5). Additionally, one of the associated SNPs was at the *Rp1* locus itself.

Expression profiling was performed to compare gene expression in the leaves of 18-day old seedlings from near-isogenic lines with and without *Rp1-D21* in B73×H95 and Mo17×H95 F1 hybrid backgrounds. Twelve of the 44 candidate genes were differentaily expressed (in all cases up-regulated in the presence of *Rp1-D21*) while 15 were not (Table 1, File S5). Unique transcripts were not detected for the remaining 17 candidate genes.

### Correlations between HR-related traits and disease resistance traits

Correlation coeffecients were estimated between line mean values for the *Rp1-D21*-associated HR-related traits and three different maize disease resistance traits (Southern leaf blight- SLB, Northern leaf blight- NLB and Grey leaf spot- GLS) previously examined on the *per se* (i.e. not crossed to *Rp1-D21-*H95) NAM populations [33]–[35]. For the correlations with disease resistance traits, analysis was performed on pooled data from 24 NAM families (all the NAM families except HP301) and the IBM population (Table 2) and on each of the 25 biparental populations separately (Table S2). The HR-related traits were all highly significantly correlated with each other with correlation coefficients over the whole NAM population ranging from 0.70 to 0.89 (Table 2). With the exception of DTAR/GLS, the HR-related traits all showed highly significant (*p*<0.0001) but low correlations (range: 0.08 to 0.15) with all the disease traits (Table 2). In all cases, enhanced HR was associated with enhanced disease resistance. Correlations analyzed within individual NAM families varied substantially between families and traits (Table S2).

**Table 2:** Correlation coefficients between mean values of NAM RILs for lesion mimic traits and disease resistance score values obtained from previous studies (SLB, Kump et al. [34]; NLB, Poland et al. [35]; GLS, Benson [33]).

Traits	*Scores on NAM populations _(n<3606)_*		
	HTR	SWR	DTAR
LES*_inv_*	0.85**** *^(n = 3576)^*	0.80**** *^(n = 3576)^*	0.65**** *^(n = 3576)^*
HTR		0.84**** *^(n = 3581)^*	0.70**** *^(n = 3582)^*
SWR			0.60**** *^(n = 3580)^*

	SLB*_inv_*	GLS	NLB
LES*_inv_*	0.06** *^(n = 3421)^*	0.08**** *^(n = 2520)^*	0.11**** *^(n = 3091)^*
HTR	0.06*** *^(n = 3423)^*	0.10**** *^(n = 2522)^*	0.13**** *^(n = 3093)^*
SWR	*ns^(n = 3423)^*	0.07*** *^(n = 2522)^*	0.11**** *^(n = 3093)^*
DTAR	0.04* *^(n = 3429)^*	0.06** *^(n = 2528)^*	0.10**** *^(n = 3098)^*

LES, lesion score; HTR, height ratio; SWR, stalk width ratio; DTAR, days to anthesis ratio; SLB, southern leaf blight resistance score ; GLS, gray leaf spot resistance score; NLB, northern leaf blight resistance score; N, sample size and *_inv_* indicates that the original lesion/disease rating scale was inverted so that the correlation sign was consistent between comparisons so that in every case, a positive correlation implies increased HR was correlated with increased HR or with increased disease resistance. Significance of correlation coefficients (*r*);

****P<0.0001,

***P*<*0.001,

**P*<*0.01,

*P*<*0.05.

ns- not significant.

The correlation between allele effect estimates for shared QTL between traits were also calculated (Table S3). Among shared QTL for HR-related traits, effects were highly correlated (range: 0.74–0.91, p<0.0001). Fourteen QTL co-localized between previously identified SLB QTL [34] and the HR-trait QTL (Figure 1). Effect estimate correlations were calculated on an individual QTL basis for each pair of colocalizing QTL. Only the QTL on chromosome 3 and 7 showed modest postive effect correlations (Table S4) while the chromosome 9 QTL effect estimates were negatively correlated, i.e. stronger HR was correlated with lower resistance. Of 11 QTL that colocalized between the HR-related QTL and NLB resistance QTL (Figure 1), only the QTL on chromosome 1 had significantly correlated effect estimates (Table S4).

## Discussion

This report comprehensively describes the genetic architecture controlling natural variation in the HR defense response in maize. We have used the MAGIC procedure [31], in which modifiers of an ectopic HR phenotype conferred by the autoactive R-gene allele *Rp1-D21* were mapped in a set of 3381 lines from the maize NAM population [32]. The modulation of wild-type HR would normally be unmeasurable since the reponse is so rapid and localized. Our approach exposes allelic variants regulating HR since the *Rp1-D21* perturbs the system at the very onset of HR initiation. The approach we have taken here lacks the ability to detect loci at which the allele inherited from the reference line (H95) is dominant to both the parental alleles from the NAM population and masks potential functional variation. This can be addressed by employing different crossing schemes [31].

The measurements used to quantify the HR by necessity conflate several different possible effects on the HR phenotype,e.g. changes in lesion number, size and shape. It is difficult to assess these separate component traits in a robust way in large segregating populations over multiple environments. In previous work we had used image analysis of a smaller segregating population to try to achieve this [27]. However the analysis was both very time consuming and yielded traits with low heritabilities, unsuitable for quantitative analysis. It is likely that the different pathways identified will affect the HR in different ways and elucidation of this will require further detailed analysis.

The MAGIC approach has been used succesfully to map modifiers of HR in other recent studies [23], [24], [36], [37]. The major advances here are that the much larger size (about 11-fold higher), diverse genetic makeup of, and the detailed genotypic data available for the NAM population, allow us for the first time to comprehensively describe the genetic architecture and to identify a set of specific genes and pathways implicated in controlling the plant HR, specifically; redox homeostasis, lignin biosynthesis, calcium signalling, programed cell death, autophagy, ubiqutin-mediated protein degradation, interaction with other R-gene paralogs.

### Correlation between disease resistance and HR severity

The heritability estimates observed for LES, HTR and SWR in the NAM population were very high (all >0.8 , Table S1) indicating a strong genetic influence on variation in these traits. The NAM population has been previously evaluated for disease resistance to SLB [34], NLB [35] and GLS [33]. Low but highly significant correlations were detected between the HR-related traits and the disease resistance traits over the entire NAM population (Table 2). Importantly, the correlations are in the same direction; increased HR was associated with increased resistance. At the level of individual QTL, the story was not as straight-forward. Of 14 and 11 QTL co-localizing between the HR-trait QTL and SLB and NLB QTL respectively only two and one respectively showed modest postive effect correlations between their allelic effects (Table S4) while the effects of an SLB QTL on chromosome 9 were negatively correlated with HR-traits, i.e. stronger HR was correlated with lower resistance. Overall it is clear that the majority of HR QTL do not coincide with a disease resistance QTL (of the three diseases assessed in NAM so far) or if they do, that the QTL have uncorrelated effects. Together with the low correlations over the entire population, this suggests the genetic control of these traits is substantially, though (importantly) not entirely, independent.

HR is mostly associated with resistance to biotrophic pathogens [38] while SLB, NLB and GLS are substantially necrotrophic [39]. We expect a stronger correlation would be found with resistance to a biotrophic disease. With this in mind, it is of note that the most biotrophic of the three diseases assessed, NLB [40] shows the highest correlations with HR. We would also expect to obtain stronger correlations if both traits had been measured in *per se* lines rather than disease resistance evaluated on *per se* lines and HR on F1 hybrids as was the case.

Necrotrophs can exploit the HR, sometimes triggering it ‘deliberately’ as part of their pathogenesis process [41], [42]. So it might seem counter-intuitive that enhanced HR is associated even at a low level with enhanced reistance rather than susceptibility to SLB and GLS in particular. However necrotrophy and biotrophy are two extremes of a continuum and the relationship between cell death and disease resistance or susceptibility is complex [43]. The causal agents of SLB and GLS (the fungi *Cochliobolus heterostrophus* and *Cercospora zeae-maydis* respectively) grow in the living plant for some time (∼1–2 days for SLB, ∼2 weeks for GLS) before any host cell death is apparent. It is likely that an HR or associated response at this ‘biotrophic’ stage of the disease would confer some resistance. The exploitation of HR by necrotrophic disease is a possible explanation for the negative effect correlations between the SLB and HR effects for the chromosome 9 QTL.

### Joint linkage and individual population analysis

The joint linkage analyses identified 32 distinct non-overlapping QTL for all traits (Figure 1). The variable effect estimates for most QTL alleles across poulations implies the presence of alleleic series at most QTL postions (Fig. 2, Fig. S2, File S2). Most of the QTL had small effects, but two QTL on chromosomes 1 (peak at ∼35 cM) and 10 (∼34.5 cM) had major contributions to phenotypic variance with *R^2^* values as high as 32 and 37%, respectively in specific populations (File S2, Fig. 2, Fig. S2). The single population analyses largely confirmed the joint-linkage analysis results with additional small effect QTL identified in specific populations. All QTL previously identified in the IBM [23], [24] and 3 NAM mapping populations [24] were detected in our current study.

The additive effects of QTL reflect their average effects over segregating genetic backgrounds, and thus across many possible epistatic interactions [44]. We did not find any QTL - QTL interactions that explained significant variation in lesion mimic-derived traits beyond what was already explained in the additive joint linkage QTL model. This result is congruent with results from other complex quantitative traits analyzed with NAM [34], [45]–[47]. Statistical epistasis is a more general concept than functional epistasis [48] and depends on allele frequencies in the study population. QTL - QTL interactions can be difficult to detect even when the genes underlying QTL interact epistatically in regulatory or biochemical pathways [44]. Thus, our inability to detect such statistical interactions does not imply that the causative genes do not interact epistatically in a functional sense.

### Genome-wide association analyses

The NAM population was specificaly designed for high-resolution dissection/fine mapping of QTL [49] as implemented with GWA analysis [34], [35], [50], [51]. Linkage disequilibrium and hence mapping resolution will vary across the genome and the closest gene to an associated SNP may not always be the causal gene [e.g. 52]. Also, it is well known that gene complements vary significantly between lines, so that the causal gene may not always be present in the sequenced B73 genome [53]. Still, previous NAM GWA studies and validation with fine mapping/positional cloning in independent near-iosgenic line (NIL) populations demonstrate resolution in some cases is sufficient to identify causative genes such as DGAT, a key determinant of oil content [50], [54] and the flowering time gene VGT1 [55], [56]. Furthermore, in this study we used a substantially more detailed genetic map than was used in these previous studies (7386 SNPs used for joint linkage compared to 1106 previously, ∼26 million used for GWA (see Materials and Methods and [57]) compared to ∼1.6 million previously) and complemented the GWA analysis with transcriptome profiling to compare gene expression between near isogenic hybrids differing for *Rp1-D21* in two hybrid backgrounds (B73×H95 and Mo17×H95).

Forty-four significantly associated SNP loci were identified (Table 1, Figure S4, File S5), 36 of which mapped to one of the QTL identifed by joint linkage analysis. The large-effect QTL on chromsome 1 and 10 harboured SNPs with lowest p values (as low as 1.89×10^−36^; Table 1, File S5) and highest RMIP (up to 0.95) and allelic effect values, indicating strong associations and high contributions to the phenotype variance. In some cases and particularly in QTL with large confidence intervals, GWA identified more than one associated genomic region within a single QTL. A previous publication [27] used the 231 lines from the maize association population [58] and identified 6 SNPs significantly associated with variation in HR (Fig. 1). Of these, the three with the largest effects were closely linked on chromosome 10 at 21.7–21.8 Mbp, in precisely the same position as the major QTL and SNP identified in this study. However, the other three SNPs identified by Olukolu et al [27], on chromosomes 5, 7 and 9, were not identified in the present study. While the 26 parents of the NAM population are a subset of the maize association population and were chosen to maximize the included diversity, a substantial amount of the diversity present in the association population is not included in the NAM population. It is possible that the causal polymorphisms underlying these SNP on chromosomes 5,7 and 9 were simply not segregating in the NAM or were segregating in only one or two populations, making their effects hard to detect. This does point out an obvious limitation in any study of this type that aims to describe the genetic architecture underlying natural variation in a trait; namely, only variation captured among the original parental lines of the population used can be detected. The NAM parental lines capture about 57% of the variation captured amongst 2,815 maize inbred accessions collected from maize breeding programs all over the world [59]. Furthermore alleles that segregate in only one or two of the 25 subpopulations that comprise the NAM population may not be detected by joint analysis or GWA due to lower power. In these cases, single population analysis may be more effective in identifying rare but important QTL. Figures 3 and S3 illustrate this point quite clearly. Two of the three SNPs identified by Olukolu et al [27] that were not identified in the present study, on chromosomes 7 and 9, were within QTL identified in individual population analyses (TX303 and NC358 families, respectively) but not in the joint analysis (File S2, S3, Fig. 3, Fig. S3).

### Candidate genes and underlying pathways

Remarkably, most of the 44 candidate genes identified are predicted to function in a discrete set of interconnected pathways associated with functions that *a priori* would seem likely to be involved in HR, including programmed cell death, defense response, ubiquitination, redox homeostasis, autophagy, calcium signalling, lignin biosynthesis and cell wall modification (Table 1). Furthermore, the expression of many of these genes were upregulated in the presence of *Rp1-D21*.

Lignification of cell walls is an important part of the HR defense response and inhibtion of lignin bisynthesis has been shown to reduce the effectiveness of HR and resistance [60], [61]. Lignin depositon is usually observed in the cells around the point of HR initiation [62]. The hydroxycinnamoyl-CoA shikimate/quinate hydroxycinnamoyl transferase (HCT) gene is the gene closest to the most highly associated SNP (RMIP = 0.95) and is predicted to be involved in lignin biosynthesis. It is the most differentially upregulated candidate gene in the *Rp1-D21*-carrying compared to the wild type hybrids in both backgrounds tested (296-fold in B73×H95 and 224-fold in Mo17×H95, Table 2). A second candidate gene, caffeoyl-CoA O-methyltransferase (CCoAOMT), is also predicted to be involved in lignin biosynthesis downstream in the pathway from HCT [63]. This gene was also significantly upregulated in NILs carrying *Rp1-D21*. The same CCoAOMT gene was also identified as a candidate gene for SLB resistance [34]. The allele effect estimates for the SLB resistance and the HR-related QTL at this locus are negatively correlated, i.e. increasing strength of HR is correlated with reduced levels of SLB resistance. This may be an example where an allele that enhances HR can be exploited by a necrotroph that can grow on dead tissue, as discussed above.

Several studies have implicated autophagy in plant immunity and HR [64]. In particular, it has been shown to be important for restricting the spread of plant HR cell death [65], [66]. The ability of cells to remove damaged organelles/cellular components and recycle them during stress and nutrient starvation is an essential rescue mechanism for cells to escape from the progression of cell death [67]. This pro-survial mechanism might be particularly important for resistance against necrotophic pathogens that promote cell death for nutrition [68], [69]. Conversely, autophagy has been implicated as a pro-death signal in HR triggered by certain R-genes in Arabidopsis [70]. These apparently contradictory roles of autophagy in plant cells have also been noted in the animal autopahagy literature [71].

Of the three candidate genes identified in this study with predicted roles in autophagy (Table 1), only the *UEV domain/VPS23/ELC* gene, located in the major QTL on chromosome 10, was significantly upregulated in NILs carrying *Rp1-D21*. The other autophagy-related candidate genes include modifier of rudimentary *(Mod(r)) protein/VPS37* and *heat shock cognate 70* (*HSC70*). Both the *UEV domain/VPS23/ELC* and *VPS37* genes (VPS stands for vacuolar protein sorting) are predicted to be involved in the biogenesis of multivesicular bodies (MVBs) and endosomal sorting of membrane cargo [72], [73]. ESCRT-I, a 350-kDa protein complex, comprises class E VPS proteins, VPS23, VPS28, and VPS37, that are required for binding and sorting of mono-ubiquitinated MVB cargoes, an interaction that seems to occur via the VPS23 subunit (a ubiquitin E2 variant) and the chaperone HSC70 [74], [75]. Damaged proteins that are targeted to the MVB membrane by VPS23 and VPS37 become internalized through the invagination of the outer membrane and finally fuse with the vacoule or lysosome where damaged proteins are degraded by the luminal proteases [72], [73].

Sixteen of the candidate genes have predicted roles in a PCD. The expression of five of these genes was upregulated in NILs carrying *Rp1-D21*. The *Rp1-D21* phenotype is more severe in the Mo17×H95 background than in the B73×H95 backgound [23]. In this context, it is interesting to note that in all five cases, the genes were more up-regulated in the Mo17×H95 background. These five candidate genes include *Spotted leaf 11* (*SPL11*)/*plant U-box 13 (PUB13)*, *RP1*/NB-ARC domain-containing disease resistance protein, Serine/threonine-protein kinase *CTR1*, Lipoxygenase 3 (*LOX3*) and a RING/U-box superfamily protein. Homologs of three of these genes are involved directly or indirectly in the induction of a spontaneous HR similar to the phenotype in this study. The *A. thaliana SPL11/PUB13* encodes a U-Box/Armadillo repeat protein with a E3 ubuqitin ligase activity and plays a role in the ubiquitination pathway. Mutations in this gene confer a spontaneous cell death phenotype and enhanced non-race-specific resistance to both *Magnaporthe grisea* and *Xanthomonas oryzae pv oryzae* [76]. Deletion of *CTR1* gene, a negative reglator of the ethylene signaling pathway, was shown to lead to amplification of the spontaneous cell death and defense phenotypes of the *A. thaliana vad1* (for vascular associated death 1) mutant [77]. The *RP1*/NB-ARC domain-containing candidate gene is a paralog of the *Rp1-D21* gene used in this study and is located in the same complex *Rp1* locus [19]. The fact that we identified a candidate in this region implies that Rp1 proteins may act as heterodimers so that the activity of one paralog is affected by the presence or absence of others. There is considerable evidence in the literature for these types of interactions [78]–[80]. A pepper ortholog of the two closely related Jacalin-like lectin domain-containing candidate genes has been shown to play a role in cell death and disease resistance in both pepper and *A. thaliana* [81].

The *LOX3* gene is predicted to be involved in the mediation of PCD as well as disruption of redox homeostasis. *LOX3* encodes an enzyme that dioxygenates unsaturated fatty acids , subsequently triggering lipoperoxidation of the cell membrane and synthesis of signaling molecules. The ensuing signaling results in structural and metabolic cellular changes in several pathopysiological conditions. The effect of the lipoperoxidation and the hydroperoxides by-product have been reported to induce pro-apoptotic conditions leading to HR cell death [82], [83]. Lipoxygenase genes have also been implicated in increased resistance to fungal pathogens [84].

Ubiquitin-mediated protein degradation pathways have recently emerged as major players in the regulation of R-gene mediated HR and plant immunity [85]. It is interesting therefore that nine of the candidate genes identified in this study as well as two other candidate genes identified in a previous study [27] have predicted roles in this pathway. Of particular interest here are examples for which the stability of canonical NBS-LRR type R-genes is regulated by ubiquitin-dependant mechanisms, conferring direct effects on R-gene function. Probably the best example of this is the case of the F-box gene *CPR1* [86], a gene that determines the specificity of the SCF ubiquitin E3 ligase complex. CPR1 targets the *R*-gene *RPS2* and the NBS-LRR R-gene homolog *SNC1* for degradation. The protein accumulation, but not the corresponding steady state transcript level of both *RPS2* and *SNC1* increase substantialy in *cpr1* mutants and are reduced in *CPR1* overexpressing lines (with a corresponding loss of immunity conferred by these genes).

Finally, three candidate genes were predicted to be involved in calcium signalling and five in redox homeostasis. Increases in cytoplasmic calcium have been associated with HR in a number of studies [87]–[89], while blockers of calcium channels and cyclic nucleotide gated channels (CNGC, the opening of which are modulated by calcium) can inhibit HR-mediated cell death [89]–[91]. The role of oxidative stess in HR is very well documented in the literature. An ‘oxidative burst’, the rapid generation of superoxide and accumulation of H_2_O_2_ is one of the first events described after elicitation of the HR [92]. The detoxification of reactive oxygen species can delay HR [e.g. 93]. Importantly calcium influx and reactive oxygen accumulation in HR appear to be mutually-dependent processes in some cases [90], [94].

In conclusion, in this study we have combined the gene-mapping power of the NAM population with the ability of the MAGIC technique to render quantifiable a previously inaccesible (though very important) phenotype. We have identified a relatively small set of candidate genes that strongly implicate a few key pathways in controlling the strength of the HR associated with *Rp1-D21*. In some cases expression analyses reinforce these conclusions. Roles for all these pathways in the control of programed cell death and HR have been demonstrated previously in the literature. While some of the loci/pathways identified may be specific to *Rp1-D21* –induced HR or to a subset of HRs induced by specific R-genes, we believe that most of are involved in HR more generally. In fact, all these pathways have been implicated in HR in other species and all were implicated by recent work that examined system-wide induced HR in tomato [95]. This work provides the most comprenensive understanding of the genetic control of the plant HR to date. Further work will attempt to dissect these effects further, to validate the genes and determine which aspect of HR, intiation, signal transduction, excecution or containment, they affect.

## Materials and Methods

### Plant materials

An *Rp1-D21*-H95 mutant line was generated from a cross between an *Rp1-D21* variant and the maize inbred line H95. The ensuing F1 progeny was then backcrossed to the H95 parent four times, while selecting for the HR phenotype marked by spontaneous lesion formation. Since the homozygous *Rp1-D21-*H95 mutant lines are sterile, it was maintained in a heterozygous state. For further details of this line see Chaikam et al [24]. The *Rp1-D21*-H95 line was crossed as a male to each of 3,381 lines from nested association mapping (NAM) population and to 225 lines from the IBM population [96] to create a set of F1 families, each of which segregated 1∶1 (wild type:mutant) for the presence and absence of the *Rp1-D21-*induced HR phenotype but which were otherwise isogenic within a family.

Development of the maize (NAM) populations has been previously described [32], [56]. This study used a subset from the NAM population that is comprised of 25 bi-parental RIL populations with B73 as a common parent and 200 lines in each population. The B73×HP301 population was not included in our panel of 3,381 lines since HP301 and about 88% of the derived lines in this population carry the *ga1*cross-incompatibility gene, making it very difficult to obtain F1 seed from these lines used as female parents [97]. The selection of 3,381 F1 families was based on the availability of seed. F1 crosses derived from the IBM population were also included in the correlation analysis part of this study but excluded from the joint linkage and genome-wide association (GWA) analysis.

### Field trials

Each of the 3,606 F1 families (including 225 IBM lines) was evaluated in four environments that comprised two locations (Clayton, NC and West Lafayette, IN) and 2 years (2011 and 2012), with single replicate of the NAM RILs in each location. An augmented randomized incomplete block design was implemented with the *Rp1-D21-*H95 as repeated check to provide an estimate of experimental error. Each NAM family was assigned to a block and a total of 10 sub-blocks within each family block. The *Rp1-D21-*H95 check was included in each of the 10 blocks in each NAM family and in each location. Two rows of a constant genotype were planted around the edges of the field to minimize border row effect. Standard fertilizer, pesticide and herbicide regimes were applied during the trial to ensure normal plant growth. Thinning to desired plant density and overhead irrigation were applied as required. At Clayton, NC, 12 kernels of each line were sown in 2 m rows with an inter-row spacing of 0.97 m and a 0.6 m alley at the end of each plot, while at West Lafayette, IN, 18 seeds were sown in 6 m rows with an inter-row spacing of 0.76 m.

### Phenotypic evaluations

Four lesion-associated traits, lesion severity (LES), mutant to wild type height ratio (HTR), mutant to wild type stalk width ratio (SWR), and mutant to wild type days to anthesis ratio (DTAR) were scored. Each F1 family segregated 1∶1 for the presence and absence of *Rp1-D21* but was otherwise isogenic. Within a family it was immediately obvious, by the presence or absence of lesions and by the growth habit of the plant, which plants carries *Rp1-D21* and which were wild-type. For the LES trait, only plants carrying *Rp1-D21* were scored, while for HTR, SWR and DTAR, both wild type and mutant classes were phenotyped separately and ratios between them derived.

#### Lesion severity (LES)

In all environments, lesion severity scores were assigned based on a scale of 1 to 10 (Figure S5), with “1” representing very few lesions and “10” indicating a completely dead plant [24]. Experiments were scored five times at Clayton, NC in both 2011 and 2012. At West Lafayette, IN, plants were scored six and four times in years 2011 and 2012, respectively. Scoring was started one month after planting and continued at approximately 10–14 day intervals.

We scored an aberrant HR defense response rather than a disease symptom in this case, but since the lesion phenotypes are generally similar to disease lesions, we applied a widely-accepted statistic in plant pathology; standardized area under disease progress curve (sAUDPC), to quantitatively measure HR severity [98]. For each environment, the sAUDPC for LES was calculated as follows: The average value of two consecutive ratings was computed and multiplied by the number of days between the ratings. Values were summed over all intervals, and then divided by the total number of days over which evaluations were performed to determine the weighted average.

#### Mutant to wild type height ratio (HTR)

Plant height data were collected after flowering from three representative mutant F1 individuals and from three representative wild type F1 individuals within each F1 family. Height means were calculated for each class within each family and the HTR was calculated by dividing the average mutant type height to the average wild type height.

#### Mutant to wild type stalk width ratio (SWR)

Stalk width immediately above the primary ear was measured on three representative mutant F1 individuals and three representative wild type F1 individuals within each F1 family. SWR was then calculated by dividing the average mutant stalk width to the wild type average stalk width.

#### Mutant to wild type days to anthesis ratio (DTAR)

Plants were monitered every other day for the date when 50% of the wild type and 50% of the mutant plants in an F1 family were shedding pollen. The days from planting to anthesis were computed for the wild type and the mutant plants in each family. DTAR was then calculated by dividing the days to anthesis for mutants by days to anthesis for wild type plants.

### Disease scores

Disease scores for SLB (southern leaf blight; *Cochliobolus heterostrophus*), GLS (gray leaf spot; *Cercospora zeae-maydis*) and NLB (northern leaf blight; *Exserohilum turcicum*) were obtained from previous studies [33]–[35]. These data were used to evaluate the correlation between disease resistance and HR severity in response to *Rp1-D21* aberrant phenotype (lesion mimics).

### Statistical analysis of phenotypic data

The least square (LS) mean data used for analysis can be found in File S6. File S7 and Figure S1 gives the metadata for each of the populations. To obtain least square mean values adjusted for environmental effects, data were analyzed with a mixed model considering line as a fixed effect and environment, block within environment, population by environment and line-by-environment interaction within population as random effects using Proc Mixed in SAS v9.3 [99]. The REML Wald's Z statistic was used to test the significance of each random factor in the model [100]. Least squares means for lines were estimated from this mixed model and used as the input phenotype for association analysis. For the purpose of estimating heritability, a mixed model with all factors, including lines, as random effects was used. Correlations between disease scores (SLB, GLS and NLB) and lesion mimic traits for each line were estimated using Proc Corr in SAS v9.3 [99]. The NAM sub-population groups, considered as a covariate, were accounted for during correlation analysis.

### Genotypic data, SNP imputation and SNP Projection

A total of 7386 SNP markers scored on all 4892 available NAM RILs were used for linkage and QTL analyses (Files S8, S9). The marker values were imputed at 0.2 cM intervals based on SNP calls made from short sequence reads using the GBS protocol [101]. Briefly, each sample was digested with the ApeK1 restriction enzyme, PCR amplified, multiplexed, sequenced and then processed through a custom SNP calling pipeline. Because the sequence coverage was low, about 0.5×, two problems arose; First, many sites had more than 50% missing data, and, second, at many heterozygous sites only a single allele of the two possible alleles was detected. As a result, about 80% of the heterozygous sites were scored as homozygous. To deal with these issues, each SNP call was first scored as either the B73 or non-B73 parent. Then the Viterbi algorithm was applied to the resulting sequence to identify probable heterozygous loci and genotype calling errors. Sites were then chosen at 0.2 cM intervals and values for each site imputed as 2 (probability allele came from the non-B73 parent) based on the nearest non-missing flanking markers. Where both flanking markers came from the same parent the value was either 0 or 2. Where the markers came from different parents, the value was intermediate and based on the relative distance from the two markers [59].

For the GWA analysis, a total of about 26.5 million SNPs polymorphic among the NAM founder lines were obtained from the HapMapv2 project [57]. The data at each locus are comprised of two alleles per SNP, with the minor alleles set to 1 and everything else to 0. The data were recoded to reduce computational time during projection by setting B73 alleles (common parental allele) to 0 and non-B73 alleles (alternate allele) to 1. Projection of the 26.5 million HapMap v2 NAM founder genotypes on the NAM RILs was performed based on the genotype of the RIL's diverse founder/parental lines at each of the 26.5 million HapMap v2 SNP, and an individual RIL's genotype calls at the NAM SNP markers flanking the physical position of the HapMap v2 SNP. Because we used the high density 7386 NAM SNP marker linkage map, each interval was only 0.2 cM in genetic distance, permitting very accurate imputation of HapMap v2 SNPs. If the diverse founder/parental line has the same homozygous allele as B73 at both flanking loci, the RIL genotype is assigned a B73 allele (coded as 0) for a SNP position. When the diverse founder/parental line carries an alternate/non-B73 allele homozygous at both flanking loci, the probability that the RIL carries the alternate allele is computed as the weighted average of the flanking mapped NAM SNP genotype, with the weights assigned based on the relative physical position of the HapMap SNP within the interval. This is computed as:
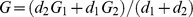
where *d*_2_ is the physical distance between the HapMap v2 SNP and the right-hand mapped NAM SNP, *G*_1_ is the genotype score at the mapped NAM SNP, *d*_1_ is the physical distance between the HapMap v2 SNP and the left-hand mapped NAM SNP, and *G*_2_ is the genotype score at the right-hand mapped NAM SNP.

### Single family and joint family linkage analysis

The single/independent population QTL analysis was performed based on composite interval mapping (CIM) and implemented in the Windows QTL Cartographer software v2.5 [102]. Permutation tests set to an alpha-level of 0.05 were performed to determine population and trait specific LOD thresholds at aprroximately 3.0. Linkage and QTL maps were prepared using the MapChart software v2.2 [103].

Joint-linkage mapping was implemented as previously described [56]. Before the joint stepwise regression procedure in PROC GLMSELECT was implemented in SAS software v9.3, LOD thresholds were established following 1,000 trait-dependent permutation tests based on alpha set at 0.05 with the model containing a family main effect. Trait specific QTL LOD thresholds of 5.44, 5.00, 4.27 and 8.34 were obtained and applied to detect QTL for LES, HTR, SWR and DTAR, respectively. The model included family main effects and a single marker effects nested within families. The lowest resulting *p* value among marker tests was obtained for each permutation.

Following initial detection of QTL loci, the stepwise regression model was optimized with an iterative process by sequentially dropping a marker in the model, testing the fit of adjacent markers until the eighth marker (1.6 cM away) from the originally selected marker, and fitting the best marker in the region back into the model. Allele effects at each marker included in the final model were estimated simultaneously using the solution option of Proc GLM in SAS software v.9.3. The *t*-tests of the null hypothesis of zero allele effect were performed for each NAM founder allele effect at each QTL. The QTL support intervals were computed in the SAS software v.9.3 by adding a single flanking marker to the full model one at a time from the QTL at a step of 0.2 cM. This was performed at the left and right side of the QTL. The support interval boundary was considered to be the last marker at which the QTL regained significance at the *p* = 0.05 level.

To test for significant digenic epistatic interactions, a subset of 1409 SNP markers were obtained from the available 7386 SNP markers at uniform 1 cM intervals and all pairwise combinations of the 1409 markers were tested separately using models that included population main effects, the two marker main effects nested in populations, and the marker-marker interaction nested in populations by extending the method of Holland [104]. The analysis was performed across the 24 NAM families while the trait-specific QTL LOD thresholds were estimated based on a permutation test at a critical value with alpha less than 5%. Marker pair interactions with *p*-values less than the permutation test-based threshold were considered for inclusion in the final joint linkage model without regard to the signficance of the main effects of the markers. Each such pair of markers and their interaction were added to the final additive joint linkage model one at a time; the *p*-value of the interaction in this full model was used to determine if the epistatic interaction improved the model fit.

### Genome-wide association analysis

GWA models were fit for each chromosome, one at a time. For each chromosome, line residual values from the final joint linkage QTL model excluding all markers on the chromosome under consideration were computed in SAS software v.9.3. Using 26.5 million SNPs, GWA (genome wide association) analysis was performed based on forward regression of the HapMap v2 SNPs to subsamples of these phenotypic residual values adjusted for QTL on other chromosomes. To identify SNPs with the most robust associations with traits, we implemented a subsampling (subagging) procedure during the GWA analysis [105], with forward regression (using a *p*-value threshold for inlcusion in the model of 1×10^−6^ [106]) performed in each of 100 subsample datasets. Each subsample dataset comprised 80% of the RILs from each NAM family [34], [51]. A population main effect was included in the model prior to the addition of SNP terms during the forward selection. The effect estimate of each significant SNP in each subsample was also computed and averaged over the 100 subsamples. The resample model inclusion probability (RMIP) was computed for each SNP as the proportion of subsample data sets in which it was included in the final regression model. Only SNPs with RMIP >0.05 are shown on the Manhattan plot. Following Valdar et al. (2006), an RMIP threshold of 0.25 was used to report the most robust SNP associations.

### Candidate gene selection

Genes co-localizing with or adjacent to associated SNPs were determined using the maize B73 reference genome assembly v2 available on the MaizeGDB genome browser [107] or the www.maizesequence.org genome browser [108]. Functional annotations of the candidate genes were determined using blastp [109], conserved domain search tools [110] and annotations avialble at the Maize Genome database (http://gbrowse.maizegdb.org/gb2/gbrowse/maize_v2/). Further annotation was achieved by inspection of the literature specific to each gene and domain (File S5).

### RNA-seq library construction, data processing and differential expressed gene analysis

Wild-type (WT) and mutant plants in B73×*Rp1-D21*-H95 and Mo17×*Rp1-D21-*H95 backgrounds growing in constant 22°C with 12 h-day/12 h-dark were used for RNA extraction. The 3rd true leaves of 5 individual seedlings collected from 18-day old WT or mutant plants were pooled and total RNA was isolated using Trizol reagent (Life Technologies) according to manufacturer's instructions. RNA concentration and quality were monitored by the NanoDrop and agarose gel electrophoresis. mRNA was isolated from the total RNA by Dynabeads oligo(dT) (Life Technologies) following manufacturer's directions. RNA-seq libraries were constructed according to the TruSeq RNA Sample Prep v2 LS protocol (Illumina) and sequenced using the Illumina HiSeq 2000. Two biological replicates, each consisting of 5 individual plants, and two technical replicates (two lanes) were performed, with 100 nt single end reads. The sequences were aligned to maize genome sequence v2 (www.maizegdb.org) via TopHat 2.0 [111], using all the default parameter settings. The raw counts per gene were calculated using in-house scripts. Only reads with unique alignments were maintained for subsequent analyses. Genes with total read counts less than 20 were filtered out. Differentially expressed genes (DEGs) were identified using the software package edgeR from the Bioconductor suite [112]. To account for multiple testing, the procedure of Benjamini and Hochberg [113] for controlling the false discovery rate (FDR) was applied using a threshold of q< = 0.05 to determine significance.

## Supporting Information

Figure S1 Distribution of least square mean values across populations shown in a bean plot.
(TIFF)

Figure S2 Heat map showing additive allelic effects for 3 HR-related traits, LES, SWR, DTAR across 24 NAM founder lines relative to the common B73 parent. Chromosome and genetic map positions (cM) of QTL peaks are shown on the left vertical axis, the contribution to phenotypic variance across all 24 NAM populations are shown on right vertical axis and the NAM founder lines are shown on the horizontal axis. Scale below heat map indicates range of allelic effect values and corresponding color intensity. Boxes with asterisks indicate significant (*p*<0.05) allelic effects.
(TIFF)

Figure S3 HTR, SWR and DTAR QTL obtained from single and joint-linkage QTL analysis across all the 10 maize chromosomes/linkage groups. Parental inbred lines crossed with the common B73 inbred line are shown on the vertical axis and represents each bi-parental mapping population. The NAM population comprising all 24 populations is indicated as JL (joint linkage analysis). The genetic distance for each chromosome is represented in cM unit on the horizontal axis.
(TIFF)

Figure S4 Results of genome-wide association analysis showing associated SNP markers above 0.05 RMIP (resample model inclusion probability). Threshold of 0.25 RMIP is indicated. Chromosomes shown on horizontal axis with SNPs in order based on physical map positions. Triangles pointing up indicate that the non-B73 allele increases the value of the trait.
(TIF)

Figure S5 Images of leaves displaying variable severities of the Rp1-D21 lesion phenotype scored on the severity scale used in this study. A 1–10 scale was used; examples are shown of leaves scored between 2 and 8 [from 25].
(TIFF)

Table S1 Heritability analyses for the traits measured in this study.
(DOCX)

Table S2 Pearson correlation coefficients between HR-related and disease traits by individual family. S2a–d correlations with LES*_inv_*, HTR, SWR, DTAR. Subscript “inv” indicates that the original lesion/disease rating scale was inverted so that the coefficient sign was consistent between comparisons so that in every case, a positive correlation implied that increased HR was correlated with increased disease resistance. Significance of correlation coefficients (r) ; ****P<0.0001, ***P<0.001, **P<0.01, *P<0.05. ns- not significant.
(DOCX)

Table S3 Correlation coefficients between QTL effect estimates across parental alleles at colocalizing QTL. n is the number of colocalizing QTL that were identified between each pair of traits. The correlation is taken between the effect estimates for each trait for each of the 24 alleles for each QTL e.g. if n = 15 , then the correlation coefficient is derived from 15×24 = 360 comparisons. ****P<0.0001.
(DOCX)

Table S4 Correlations between effect estimates at specific QTL which colocalize between HR-related trait QTL and previously identified QTL for SLB and NLB resistance. Subscript “inv” indicates that the original lesion/disease rating scale was inverted so that the coefficient sign was consistent between comparisons so that in every case, a positive correlation implied that increased HR was correlated with increased HR or disease resistance. Significance of correlation coefficients (r) ; ****P<0.0001, ***P<0.001, **P<0.01, *P<0.05, ^#^P<0.1. ns- not significant.
(DOCX)

File S1 QTL genetic and physical map 95% support interval for LES_HTR_SWR_DTAR.
(TXT)

File S2 QTL allelic effects for each NAM population.
(XLSX)

File S3 QTL peak, LOD scores, proportion of contribution to phenotypic variance (R^2^) and allelic effects of QTL computed from the single family QTL analysis on LES, HTR, SWR and DTAR.
(XLSX)

File S4 Significant SNPs at thresholds of p = 1×10^−6^ and RMIP = 0.05 for all four traits.
(TXT)

File S5 Table of candidate genes and functional annotations with more comprehensive details than shown in Table 2. Included here are effect estimates and more detailed notes on implicated pathways.
(XLSX)

File S6 LSmeans for LES, HTR, SWR and DTAR and BLUPs for SLB, GLS and NLB.
(TXT)

File S7 Sample size, mean and range of values (minimum and maximum) of traits for each NAM population.
(TXT)

File S8 Genotypes of the NAM RILs at 7386 SNP loci.
(ZIP)

File S9 Genetic linkage map of the NAM population used in this analysis.
(TXT)

File S10 Joint family QTL analysis showing loci with epistatic interactions.
(XLSX)
