# Supplementary figures and images for: A Genome-Wide Association Study of the Maize Hypersensitive Defense Response Identifies Genes That Cluster in Related Pathways
Source: PLoS Genet. 2014 Aug 28;10(8):e1004562. doi: 10.1371/journal.pgen.1004562 (PMC4148229; doi:10.1371/journal.pgen.1004562)

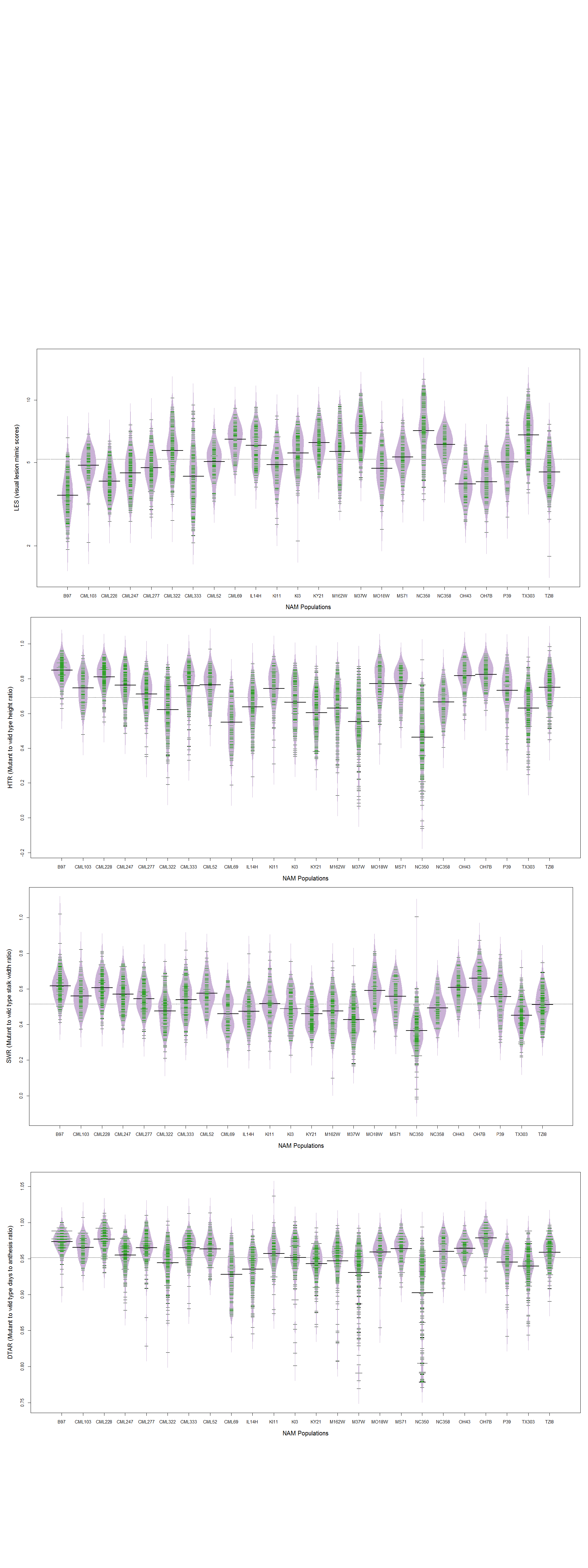

Supplement: Figure S1 — Distribution of least square mean values across populations shown in a bean plot. (TIFF) [file pgen.1004562.s001.tiff]

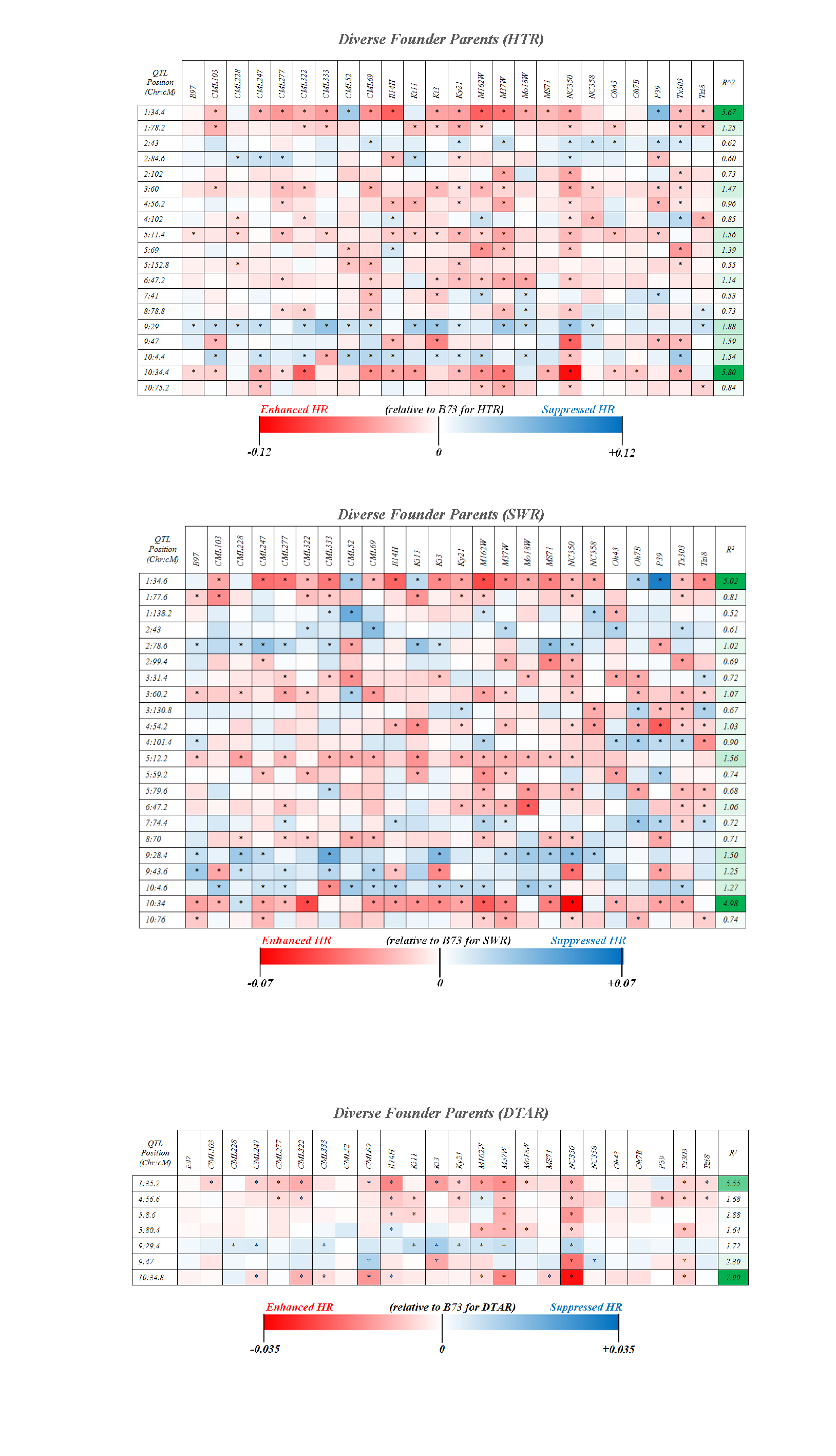

Supplement: Figure S2 — Heat map showing additive allelic effects for 3 HR-related traits, LES, SWR, DTAR across 24 NAM founder lines relative to the common B73 parent. Chromosome and genetic map positions (cM) of QTL peaks are shown on the left vertical axis, the contribution to phenotypic variance across all 24 NAM populations are shown on right vertical axis and the NAM founder lines are shown on the horizontal axis. Scale below heat map indicates range of allelic effect values and corresponding color intensity. Boxes with asterisks indicate significant (p<0.05) allelic effects. (TIFF) [file pgen.1004562.s002.tiff]

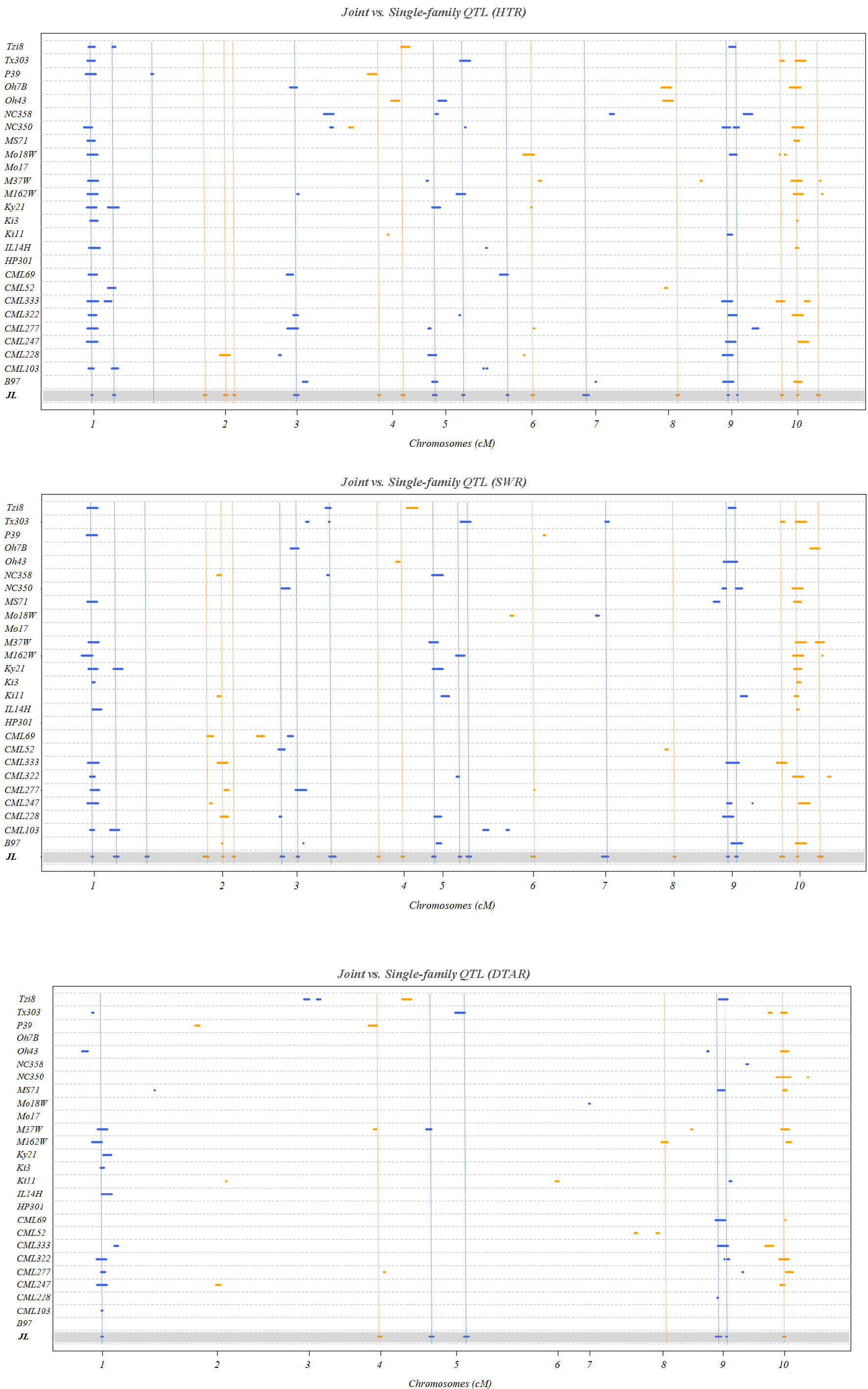

Supplement: Figure S3 — HTR, SWR and DTAR QTL obtained from single and joint-linkage QTL analysis across all the 10 maize chromosomes/linkage groups. Parental inbred lines crossed with the common B73 inbred line are shown on the vertical axis and represents each bi-parental mapping population. The NAM population comprising all 24 populations is indicated as JL (joint linkage analysis). The genetic distance for each chromosome is represented in cM unit on the horizontal axis. (TIFF) [file pgen.1004562.s003.tiff]

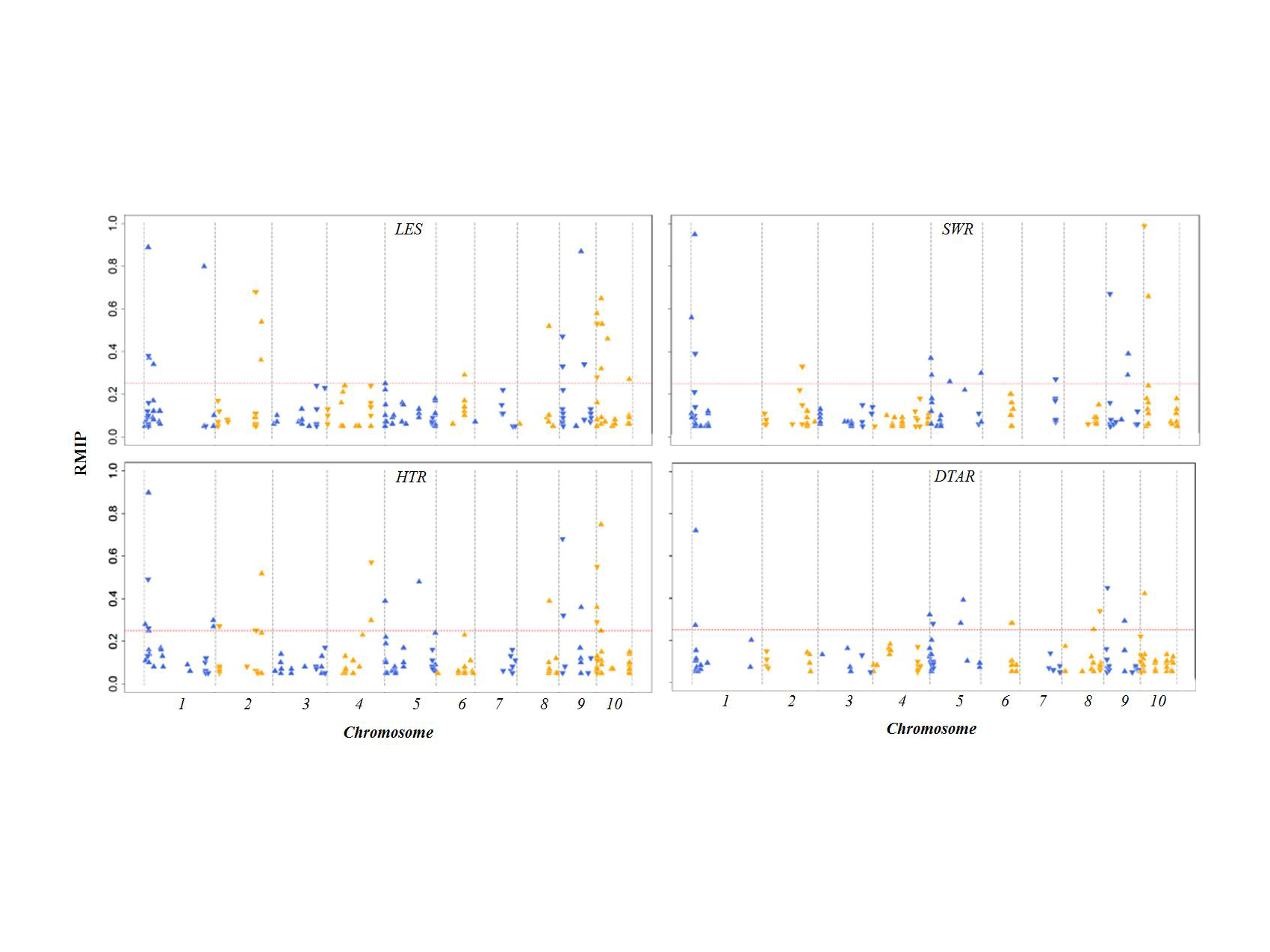

Supplement: Figure S4 — Results of genome-wide association analysis showing associated SNP markers above 0.05 RMIP (resample model inclusion probability). Threshold of 0.25 RMIP is indicated. Chromosomes shown on horizontal axis with SNPs in order based on physical map positions. Triangles pointing up indicate that the non-B73 allele increases the value of the trait. (TIF) [file pgen.1004562.s004.tif]

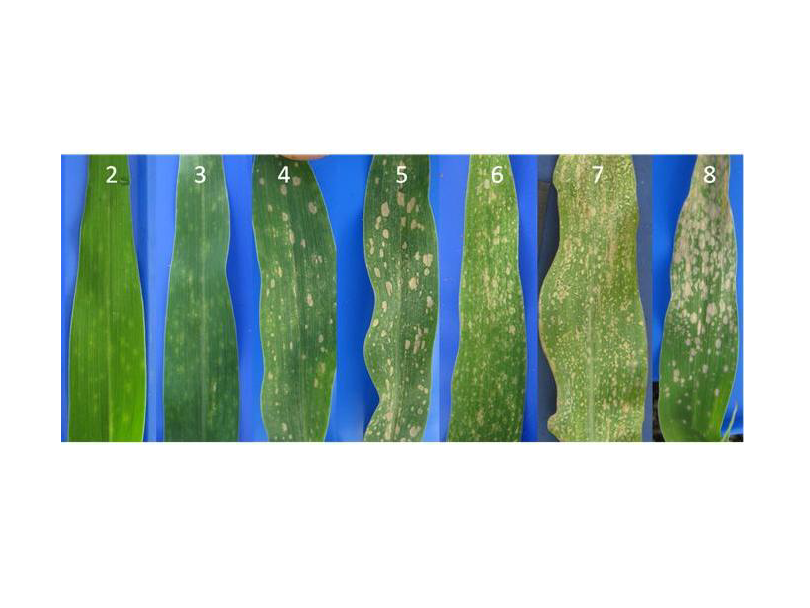

Supplement: Figure S5 — Images of leaves displaying variable severities of the Rp1-D21 lesion phenotype scored on the severity scale used in this study. A 1–10 scale was used; examples are shown of leaves scored between 2 and 8 [from 25]. (TIFF) [file pgen.1004562.s005.tiff]
