# Supplementary material for: A Genome-Wide Association Study of the Maize Hypersensitive Defense Response Identifies Genes That Cluster in Related Pathways
Source: PLoS Genet. 2014 Aug 28;10(8):e1004562. doi: 10.1371/journal.pgen.1004562 (PMC4148229; doi:10.1371/journal.pgen.1004562)
Supplement: Table S1 — Heritability analyses for the traits measured in this study. (DOCX) [file pgen.1004562.s006.docx]

Table S1:

| Phenotype | Heritability | |
| --- | --- | --- |
|  | Plot-Basis | Line Mean-Basis |
| LES | 0.639 | 0.874 |
| HTR | 0.648 | 0.877 |
| SWR | 0.560 | 0.830 |
| DTAR | 0.268 | 0.584 |

Note: LES - Lesion score, HTR - Height ratio, SWR - Stalk width ratio, DTAR – Days to anthesis ratio.
