# Supplementary material for: A Genome-Wide Association Study of the Maize Hypersensitive Defense Response Identifies Genes That Cluster in Related Pathways
Source: PLoS Genet. 2014 Aug 28;10(8):e1004562. doi: 10.1371/journal.pgen.1004562 (PMC4148229; doi:10.1371/journal.pgen.1004562)
Supplement: Table S2 — Pearson correlation coefficients between HR-related and disease traits by individual family. S2a–d correlations with LESinv, HTR, SWR, DTAR. Subscript “inv” indicates that the original lesion/disease rating scale was inverted so that the coefficient sign was consistent between comparisons so that in every case, a positive correlation implied that increased HR was correlated with increased disease resistance. Significance of correlation coefficients (r) ; ****P<0.0001, ***P<0.001, **P<0.01, *P<0.05. ns- not significant. (DOCX) [file pgen.1004562.s007.docx]

Table S2a. Correlations between LES and disease traits

| Population | Founder line | SLB*_inv_* | | GLS | | NLB | |
| --- | --- | --- | --- | --- | --- | --- | --- |
|  |  | *r^p-value^* | *_N_* | *r^p-value^* | *_N_* | *r^p-value^* | *_N_* |
| 1 | B97 | *^ns^* | *_182_* | *^ns^* | *_139_* | 0.16^*^ | *_172_* |
| 2 | CML103 | *^ns^* | *_96_* | *^ns^* | *_72_* | *^ns^* | *_89_* |
| 3 | CML228 | *^ns^* | *_188_* | *^ns^* | *_136_* | *^ns^* | *_151_* |
| 4 | CML247 | *^ns^* | *_168_* | 0.21^*^ | *_126_* | *^ns^* | *_151_* |
| 5 | CML277 | *^ns^* | *_166_* | 0.24^**^ | *_122_* | *^ns^* | *_134_* |
| 6 | CML322 | *^ns^* | *_158_* | *^ns^* | *_123_* | 0.22^**^ | *_144_* |
| 7 | CML333 | -0.27^***^ | *_166_* | -0.21^*^ | *_128_* | *^ns^* | *_155_* |
| 8 | CML52 | *^ns^* | *_90_* | *^ns^* | *_68_* | *^ns^* | *_75_* |
| 9 | CML69 | *^ns^* | *_97_* | *^ns^* | *_71_* | *^ns^* | *_87_* |
| 10 | HP301 | *_._* | *_._* | *_._* | *_._* | *_._* | *_._* |
| 11 | IL14H | 0.22^**^ | *_142_* | *^ns^* | *_107_* | *^ns^* | *_134_* |
| 12 | KI11 | *^ns^* | *_94_* | *^ns^* | *_67_* | *^ns^* | *_77_* |
| 13 | KI3 | *^ns^* | *_121_* | 0.19^*^ | *_113_* | 0.27^**^ | *_117_* |
| 14 | KY21 | 0.21^**^ | *_186_* | 0.28^**^ | *_94_* | *^ns^* | *_178_* |
| 15 | M162W | *^ns^* | *_162_* | *^ns^* | *_127_* | 0.23^**^ | *_154_* |
| 16 | M37W | *^ns^* | *_190_* | *^ns^* | *_144_* | 0.22^**^ | *_177_* |
| 17 | MO17 | *^ns^* | *_125_* | 0.27^*^ | *_86_* | *^ns^* | *_102_* |
| 18 | MO18W | *^ns^* | *_95_* | *^ns^* | *_71_* | 0.25^*^ | *_85_* |
| 19 | MS71 | *^ns^* | *_97_* | *^ns^* | *_75_* | *^ns^* | *_95_* |
| 20 | NC350 | 0.24^***^ | *_185_* | 0.16^*^ | *_139_* | 0.20^*^ | *_168_* |
| 21 | NC358 | *^ns^* | *_85_* | *^ns^* | *_65_* | *ns* | *_77_* |
| 22 | OH43 | *^ns^* | *_92_* | *^ns^* | *_73_* | 0.27^*^ | *_86_* |
| 23 | OH7B | *^ns^* | *_92_* | *^ns^* | *_70_* | *^ns^* | *_79_* |
| 24 | P39 | *^ns^* | *_85_* | *^ns^* | *_67_* | *^ns^* | *_75_* |
| 25 | TX303 | *^ns^* | *_190_* | *^ns^* | *_145_* | *^ns^* | *_177_* |
| 26 | TZI8 | *^ns^* | *_169_* | *^ns^* | *_92_* | *^ns^* | *_152_* |
|  | NAM | 0.06^**^ | *_3421_* | 0.08^****^ | *_2520_* | 0.11^****^ | *_3091_* |

Table S2b Correlations between HTR and disease traits:

| Population | Founder line | SLB*_inv_* | | GLS | | NLB | |
| --- | --- | --- | --- | --- | --- | --- | --- |
|  |  | ***r^p-value^*** | ***_N_*** | ***r^p-value^*** | ***_N_*** | ***r^p-value^*** | ***_N_*** |
| 1 | B97 | *^ns^* | *_182_* | *^ns^* | *_139_* | 0.17^*^ | *_172_* |
| 2 | CML103 | *^ns^* | *_96_* | *^ns^* | *_72_* | *^ns^* | *_89_* |
| 3 | CML228 | *^ns^* | *_188_* | *^ns^* | *_136_* | 0.21^**^ | *_151_* |
| 4 | CML247 | *^ns^* | *_168_* | *^ns^* | *_126_* | 0.22^**^ | *_151_* |
| 5 | CML277 | 0.18^*^ | *_166_* | 0.24^**^ | *_122_* | *^ns^* | *_134_* |
| 6 | CML322 | *^ns^* | *_158_* | *^ns^* | *_123_* | 0.25^**^ | *_144_* |
| 7 | CML333 | -0.23^**^ | *_166_* | *^ns^* | *_128_* | *^ns^* | *_155_* |
| 8 | CML52 | *^ns^* | *_90_* | *^ns^* | *_68_* | *^ns^* | *_75_* |
| 9 | CML69 | 0.21^*^ | *_97_* | *^ns^* | *_71_* | *^ns^* | *_87_* |
| 10 | HP301 | *_._* | *_._* | *_._* | *_._* | *_._* | *_._* |
| 11 | IL14H | *^ns^* | *_142_* | *^ns^* | *_107_* | 0.25^**^ | *_134_* |
| 12 | KI11 | *^ns^* | *_96_* | *^ns^* | *_69_* | *^ns^* | *_79_* |
| 13 | KI3 | *^ns^* | *_121_* | *^ns^* | *_113_* | 0.22^*^ | *_117_* |
| 14 | KY21 | 0.19^**^ | *_186_* | 0.23^*^ | *_94_* | 0.14^*^ | *_178_* |
| 15 | M162W | *^ns^* | *_162_* | *^ns^* | *_127_* | 0.25^**^ | *_154_* |
| 16 | M37W | *^ns^* | *_190_* | *^ns^* | *_144_* | 0.22^**^ | *_177_* |
| 17 | MO17 | *^ns^* | *_125_* | 0.24^*^ | *_87_* | *ns* | *_103_* |
| 18 | MO18W | *^ns^* | *_95_* | *^ns^* | *_71_* | 0.23^*^ | *_85_* |
| 19 | MS71 | *^ns^* | *_96_* | *^ns^* | *_74_* | *^ns^* | *_94_* |
| 20 | NC350 | 0.24^**^ | *_186_* | 0.18^*^ | *_140_* | 0.17^*^ | *_169_* |
| 21 | NC358 | *^ns^* | *_85_* | *^ns^* | *_65_* | *^ns^* | *_77_* |
| 22 | OH43 | *^ns^* | *_92_* | *^ns^* | *_73_* | 0.23^*^ | *_86_* |
| 23 | OH7B | *^ns^* | *_92_* | *^ns^* | *_70_* | *^ns^* | *_79_* |
| 24 | P39 | *^ns^* | *_86_* | *^ns^* | *_68_* | *^ns^* | *_76_* |
| 25 | TX303 | *^ns^* | *_189_* | 0.19^*^ | *_145_* | *^ns^* | *_176_* |
| 26 | TZI8 | *^ns^* | *_170_* | *^ns^* | *_92_* | *^ns^* | *_153_* |
|  | NAM | 0.06^***^ | *_3423_* | 0.10^****^ | *_2522_* | 0.13^****^ | *_3093_* |

Table S2c Correlations between SWR and disease traits:

| Population | Founder line | SLB*_inv_* | | GLS | | NLB | |
| --- | --- | --- | --- | --- | --- | --- | --- |
|  |  | ***r^p-value^*** | ***_N_*** | ***r^p-value^*** | ***_N_*** | ***r^p-value^*** | ***_N_*** |
| 1 | B97 | *^ns^* | *_182_* | *^ns^* | *_139_* | *^ns^* | *_172_* |
| 2 | CML103 | *^ns^* | *_96_* | *^ns^* | *_72_* | *^ns^* | *_89_* |
| 3 | CML228 | *^ns^* | *_188_* | *^ns^* | *_136_* | *^ns^* | *_151_* |
| 4 | CML247 | *^ns^* | *_168_* | *^ns^* | *_126_* | 0.16^*^ | *_151_* |
| 5 | CML277 | *^ns^* | *_166_* | *^ns^* | *_122_* | *^ns^* | *_134_* |
| 6 | CML322 | *^ns^* | *_158_* | *^ns^* | *_123_* | 0.18^*^ | *_144_* |
| 7 | CML333 | -0.33^****^ | *_166_* | *^ns^* | *_128_* | *^ns^* | *_155_* |
| 8 | CML52 | *^ns^* | *_90_* | *^ns^* | *_68_* | *^ns^* | *_75_* |
| 9 | CML69 | *^ns^* | *_97_* | *^ns^* | *_71_* | *^ns^* | *_87_* |
| 10 | HP301 | *_._* | *_._* | *_._* | *_._* | *_._* | *_._* |
| 11 | IL14H | 0.22^**^ | *_142_* | *^ns^* | *_107_* | 0.23^**^ | *_134_* |
| 12 | KI11 | *^ns^* | *_96_* | *^ns^* | *_69_* | *^ns^* | *_79_* |
| 13 | KI3 | *^ns^* | *_122_* | *^ns^* | *_114_* | *^ns^* | *_118_* |
| 14 | KY21 | 0.16^*^ | *_186_* | 0.28^**^ | *_94_* | *^ns^* | *_178_* |
| 15 | M162W | *^ns^* | *_162_* | *^ns^* | *_127_* | 0.22^**^ | *_154_* |
| 16 | M37W | *^ns^* | *_190_* | *^ns^* | *_144_* | 0.14^*^ | *_177_* |
| 17 | MO17 | *^ns^* | *_125_* | 0.25^*^ | *_87_* | ns | *_103_* |
| 18 | MO18W | -0.21^*^ | *_95_* | *^ns^* | *_71_* | 0.34^**^ | *_85_* |
| 19 | MS71 | *^ns^* | *_96_* | *^ns^* | *_74_* | *ns* | *_94_* |
| 20 | NC350 | 0.22^**^ | *_185_* | 0.23^**^ | *_139_* | 0.21^**^ | *_168_* |
| 21 | NC358 | *^ns^* | *_85_* | *^ns^* | *_65_* | *^ns^* | *_77_* |
| 22 | OH43 | *^ns^* | *_92_* | *^ns^* | *_73_* | 0.32^**^ | *_86_* |
| 23 | OH7B | *^ns^* | *_92_* | *^ns^* | *_70_* | *^ns^* | *_79_* |
| 24 | P39 | *^ns^* | *_85_* | *^ns^* | *_67_* | *^ns^* | *_75_* |
| 25 | TX303 | *^ns^* | *_189_* | 0.19^*^ | *_145_* | *^ns^* | *_176_* |
| 26 | TZI8 | *^ns^* | *_170_* | *^ns^* | *_92_* | *^ns^* | *_153_* |
|  | NAM | *^ns^* | *_3423_* | 0.08^****^ | *_2522_* | 0.12^****^ | *_3093_* |

Table S2d Correlations between DTAR and disease traits:

| Population | Founder line | SLB*_inv_* | | GLS | | NLB | |
| --- | --- | --- | --- | --- | --- | --- | --- |
|  |  | ***r^p-value^*** | ***_N_*** | ***r^p-value^*** | ***_N_*** | ***r^p-value^*** | ***_N_*** |
| 1 | B97 | *^ns^* | *_182_* | *^ns^* | *_139_* | *^ns^* | *_172_* |
| 2 | CML103 | *^ns^* | *_96_* | *^ns^* | *_72_* | 0.35^***^ | *_89_* |
| 3 | CML228 | *^ns^* | *_188_* | *^ns^* | *_136_* | *^ns^* | *_151_* |
| 4 | CML247 | *^ns^* | *_168_* | *^ns^* | *_126_* | *^ns^* | *_151_* |
| 5 | CML277 | *^ns^* | *_166_* | *^ns^* | *_122_* | *^ns^* | *_134_* |
| 6 | CML322 | *^ns^* | *_158_* | *^ns^* | *_123_* | 0.25^**^ | *_144_* |
| 7 | CML333 | -0.19^*^ | *_166_* | -0.19^*^ | *_128_* | *^ns^* | *_155_* |
| 8 | CML52 | *^ns^* | *_90_* | *^ns^* | *_68_* | *^ns^* | *_75_* |
| 9 | CML69 | *^ns^* | *_97_* | *^ns^* | *_71_* | *^ns^* | *_87_* |
| 10 | HP301 | *_._* | *_._* | *_._* | *_._* | *_._* | *_._* |
| 11 | IL14H | *^ns^* | *_142_* | *^ns^* | *_107_* | *^ns^* | *_134_* |
| 12 | KI11 | -0.34^***^ | *_96_* | -0.25^*^ | *_69_* | *^ns^* | *_79_* |
| 13 | KI3 | *^ns^* | *_121_* | *^ns^* | *_113_* | *^ns^* | *_117_* |
| 14 | KY21 | *^ns^* | *_186_* | *^ns^* | *_94_* | *^ns^* | *_178_* |
| 15 | M162W | *^ns^* | *_163_* | *^ns^* | *_127_* | *^ns^* | *_154_* |
| 16 | M37W | *^ns^* | *_190_* | *^ns^* | *_144_* | *^ns^* | *_177_* |
| 17 | MO17 | *^ns^* | *_125_* | 0.29^**^ | *_86_* | *^ns^* | *_102_* |
| 18 | MO18W | -0.22^*^ | *_95_* | *^ns^* | *_71_* | 0.24^*^ | *_85_* |
| 19 | MS71 | *^ns^* | *_96_* | *^ns^* | *_74_* | *^ns^* | *_94_* |
| 20 | NC350 | 0.19^*^ | *_186_* | *^ns^* | *_140_* | 0.21^**^ | *_169_* |
| 21 | NC358 | *^ns^* | *_86_* | *^ns^* | *_66_* | *^ns^* | *_78_* |
| 22 | OH43 | *^ns^* | *_92_* | *^ns^* | *_73_* | *^ns^* | *_86_* |
| 23 | OH7B | *^ns^* | *_92_* | *^ns^* | *_70_* | *^ns^* | *_79_* |
| 24 | P39 | *^ns^* | *_87_* | *^ns^* | *_69_* | *^ns^* | *_77_* |
| 25 | TX303 | *^ns^* | *_189_* | *^ns^* | *_145_* | *^ns^* | *_176_* |
| 26 | TZI8 | *^ns^* | *_171_* | *^ns^* | *_93_* | *^ns^* | *_154_* |
|  | NAM | 0.04^*^ | *_3429_* | 0.06^**^ | *_2528_* | 0.10^****^ | *_3098_* |
