# Supplementary material for: A Genome-Wide Association Study of the Maize Hypersensitive Defense Response Identifies Genes That Cluster in Related Pathways
Source: PLoS Genet. 2014 Aug 28;10(8):e1004562. doi: 10.1371/journal.pgen.1004562 (PMC4148229; doi:10.1371/journal.pgen.1004562)
Supplement: Table S3 — Correlation coefficients between QTL effect estimates across parental alleles at colocalizing QTL. n is the number of colocalizing QTL that were identified between each pair of traits. The correlation is taken between the effect estimates for each trait for each of the 24 alleles for each QTL e.g. if n = 15 , then the correlation coefficient is derived from 15×24 = 360 comparisons. ****P<0.0001. (DOCX) [file pgen.1004562.s008.docx]

**Table S3**

| Traits |  | *QTL allele effect values* | | |
| --- | --- | --- | --- | --- |
|  |  | HTR | SWR | DTAR |
| LES*_inv_* |  | 0.91^****^  *^(n=15)^* | 0.84^****^  *^(n=14)^* | 0.82^****^  *^(n=6)^* |
| HTR |  |  | 0.87^****^  *^(n=14)^* | 0.87^****^  *^(n=6)^* |
| SWR |  |  |  | 0.74^****^  *^(n=7)^* |
