# Supplementary material for: A Genome-Wide Association Study of the Maize Hypersensitive Defense Response Identifies Genes That Cluster in Related Pathways
Source: PLoS Genet. 2014 Aug 28;10(8):e1004562. doi: 10.1371/journal.pgen.1004562 (PMC4148229; doi:10.1371/journal.pgen.1004562)
Supplement: Table S4 — Correlations between effect estimates at specific QTL which colocalize between HR-related trait QTL and previously identified QTL for SLB and NLB resistance. Subscript “inv” indicates that the original lesion/disease rating scale was inverted so that the coefficient sign was consistent between comparisons so that in every case, a positive correlation implied that increased HR was correlated with increased HR or disease resistance. Significance of correlation coefficients (r) ; ****P<0.0001, ***P<0.001, **P<0.01, *P<0.05, #P<0.1. ns- not significant. (DOCX) [file pgen.1004562.s009.docx]

**Table S4**.

| Traits |  | QTL _(_*_Chr:cM_*_)_ | SLB*_inv_* | NLB |
| --- | --- | --- | --- | --- |
| LES*_inv_* |  | *3:96.2^1^* | 0.51^***^ | - |
|  |  | *9:28.8^2^* | -0.60^**^ | *-* |
| HTR |  | *9:29.0^2^* | -0.61^**^ | - |
| SWR |  | *1:34.6* | - | 0.44^*^ |
|  |  | *9:28.4^2^* | -0.51^*^ |  |
|  |  | *7:74.4* | 0.40^*^ | 0.35^#^ |
| DTAR |  | - | - | - |
|  |  | - | - | - |
